# Anti-inflammatory reprogramming of microglia cells by metabolic modulators to counteract neurodegeneration; a new role for Ranolazine

**DOI:** 10.1038/s41598-023-47540-8

**Published:** 2023-11-17

**Authors:** Ilaria Piano, Arianna Votta, Patrizia Colucci, Francesca Corsi, Sara Vitolo, Chiara Cerri, Dario Puppi, Michele Lai, José Fernando Maya-Vetencourt, Massimiliano Leigheb, Chiara Gabellini, Elisabetta Ferraro

**Affiliations:** 1 Department of Pharmacy, University of Pisa, Pisa, Italy; 2 Department of Biology, University of Pisa, Pisa, Italy; 3 Department of Chemistry and Industrial Chemistry, University of Pisa, Pisa, Italy; 4 Retrovirus Center, Department of Translational Research and New Technologies in Medicine and Surgery, University of Pisa, Pisa, Italy; 5 Centre for Synaptic Neuroscience, Italian Institute of Technology (IIT), Genova, Italy; 6 Orthopaedics and Traumatology Unit, “Maggiore della Carità” Hospital, Department of Health Sciences, University of Piemonte Orientale (UPO), Novara, Italy

**Keywords:** Neurological disorders, Cell biology, Immunology, Neuroscience, Neurology

## Abstract

Microglia chronic activation is a hallmark of several neurodegenerative diseases, including the retinal ones, possibly contributing to their etiopathogenesis. However, some microglia sub-populations have anti-inflammatory and neuroprotective functions, thus making arduous deciphering the role of these cells in neurodegeneration. Since it has been proposed that functionally different microglia subsets also rely on different metabolic routes, we hypothesized that modulating microglia metabolism might be a tool to enhance their anti-inflammatory features. This would have a preventive and therapeutic potential in counteracting neurodegenerative diseases. For this purpose, we tested various molecules known to act on cell metabolism, and we revealed the anti-inflammatory effect of the FDA-approved piperazine derivative Ranolazine on microglia cells, while confirming the one of the flavonoids Quercetin and Naringenin, both in vitro and in vivo. We also demonstrated the synergistic anti-inflammatory effect of Quercetin and Idebenone, and the ability of Ranolazine, Quercetin and Naringenin to counteract the neurotoxic effect of LPS-activated microglia on 661W neuronal cells. Overall, these data suggest that using the selected molecules -also in combination therapies- might represent a valuable approach to reduce inflammation and neurodegeneration while avoiding long term side effects of corticosteroids.

## Introduction

Microglia are the primary innate immune effector cells of the central nervous system (CNS) mediating neuroinflammation. The primary function of microglia, once activated from their homeostatic state, is to fight pathogenic infections or to detect and counteract neural tissue damage by producing inflammatory cytokines and reactive oxygen species (ROS)^1^. However, an excessively stimulated microglia immune response might become harmful to the CNS and directly contribute to the pathogenesis of several neurodegenerative diseases, including various retinal dystrophies. In fact, neuroinflammation and microglia activation are early common features among many retinal neurodegenerative disorders, including age macular degeneration, retinitis pigmentosa and diabetic retinopathy.

Since immune activation may have deleterious consequences to the surrounding tissue, microglia are normally characterized by mechanisms counterbalancing inflammation and promoting their homeostatic state. Along this line, microglia have also been associated with reparative processes as they, by releasing anti-inflammatory cytokines and growth factors, can exhibit neuroprotective functions, thus preventing mitochondrial damage and neurodegeneration^2–4^. These apparently contradictory roles of microglia might be explained by the presence of multiple distinct microglial subpopulations having different molecular signatures and functions, among which pro-inflammatory/toxic and anti-inflammatory/protective ones; indeed, microglia exist in a multitude of dynamic states constantly interchanging and not restricted to this simple dichotomy^5–9^.

Besides their different sensome and secretome, different microglia phenotypes are also associated with distinct metabolic pathways, and metabolic reprogramming occurring during microglia activation likely contributes to neuroinflammation modulation^10^. Based on these differences, it is conceivable to hypothesize that manipulating microglia metabolism might influence microglia maturation. Since glycolytic metabolism has been suggested to mainly characterize pro-inflammatory subsets, whereas a mitochondrial oxidative metabolism seems to be mainly associated to anti-inflammatory species, it has been proposed that stimulating the mitochondrial metabolism would lead to anti-inflammatory maturation, thus counteracting the pro-inflammatory one as a tool to reduce inflammation^10, 11^.

Among the molecules able to impact on cell metabolism, the piperazine derivatives Trimetazidine (TMZ) and Ranolazine (RAN) are established FDA-approved antianginal drugs being metabolic modulators partially inhibiting β-oxidation, thus favoring glucose as substrate; glucose allows a more efficient utilization of the available oxygen, thus increasing metabolic efficiency and counteracting mitochondrial dysfunctions^12–15^. TMZ and RAN have been found to increase mitochondrial protein levels and oxidative metabolism in aged and atrophic muscles^16–21^*.* Riboflavin (RBF) (also known as Vitamin B2) is crucial for mitochondrial oxidative phosphorylation and ATP generation. Inside the cells, RBF is phosphorylated to flavin mononucleotide (FMN) and flavin adenine dinucleotide (FAD) which are cofactors of hundreds of apo-flavoenzymes, among which subunits of respiratory chain complexes^22^*.* It has been found that supraphysiological doses of RBF improve energy production upon hypoxia and are beneficial for patients with mitochondrial oxidative phosphorylation dysfunction^23–25^. Idebenone (IDB) is an analogue of coenzyme Q10, a component of the electron transport chain mediating mitochondrial respiration. Also, many flavonoids have been suggested to modulate the metabolism and to activate mitochondrial biogenesis and respiration^26–28^*.* Among them, Quercetin (QUE) and Naringenin (NAR) are plant derivative flavonoids having antioxidant and anti-inflammatory effects^29–36^. The antioxidant effect of QUE is thought to be at the basis of its pro-mitochondrial metabolism effect^34, 37^*.* The action of NAR on mitochondrial metabolism and biogenesis is controversial, as some authors propose that it protects mitochondria by attenuating ROS production through GSH and Nrf2 (Nuclear factor erythroid 2-related factor 2, also known as nuclear factor erythroid-derived 2-like 2 or NFE2L2), thereby inhibiting mitochondrial oxidative stress damage^38, 39^. Other authors suggest that NAR is able to enhance mitochondrial biogenesis possibly via the AMPK-SIRT3 signaling^40^. However, their mechanism of action remains to be fully elucidated.

Here we set out to clarify how these molecules -known to modulate mitochondrial metabolism- are able to counteract the pro-inflammatory activation of microglia; in addition, we evaluated, in vitro, the relevance of this anti-inflammatory role in the context of retinal neurodegeneration, regardless of the genetic or idiopathic nature of the disease.

## Results

### Quercetin, Naringenin and Ranolazine, but not Trimetazidine and Riboflavine, have an anti-inflammatory effect on BV2 cells

**Figure 1:**
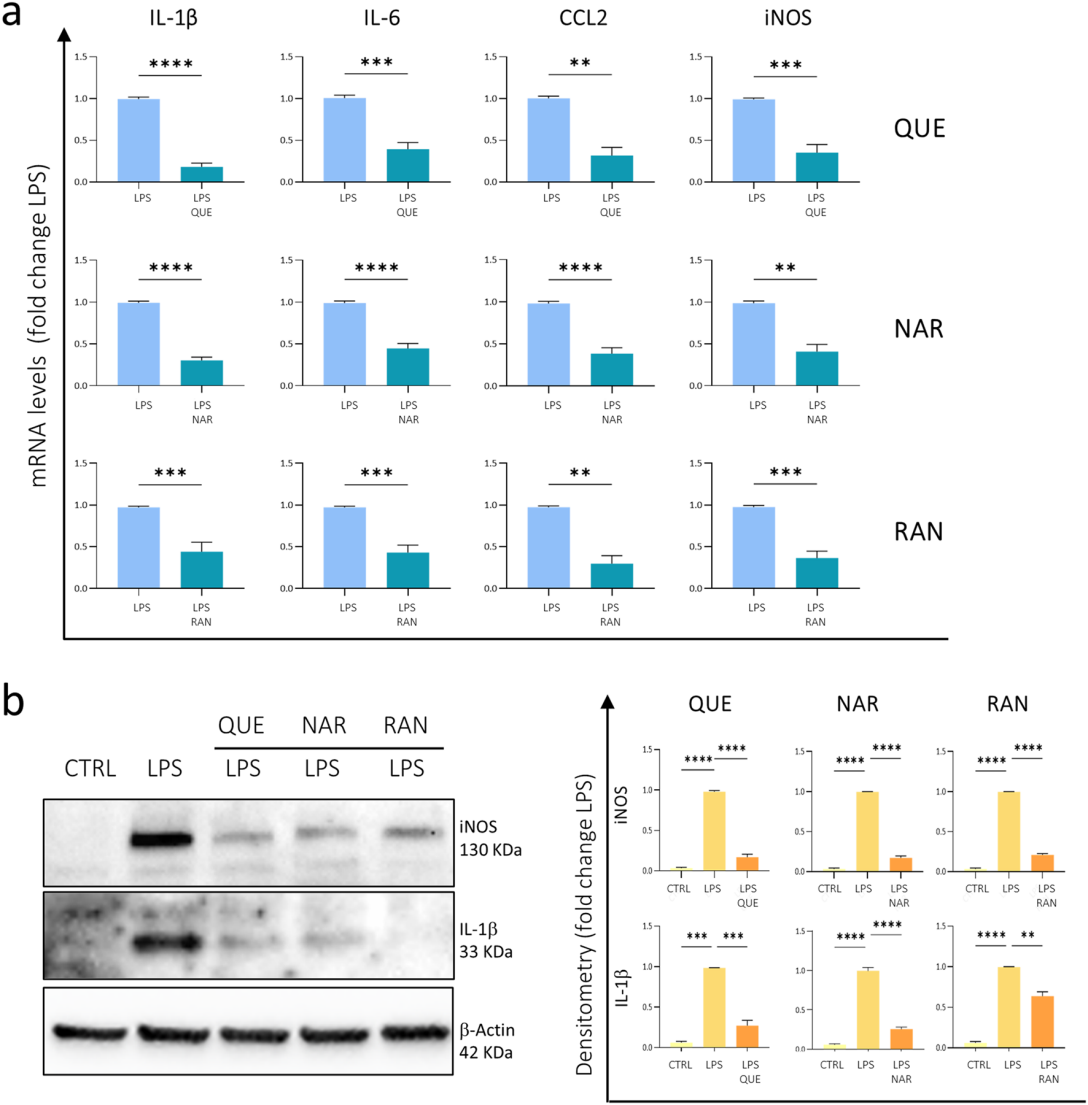
Anti-inflammatory effect of QUE, NAR and RAN on BV2 cells. (**A**) The mRNA levels of IL-1β, IL-6, CCL2 and iNOS were evaluated by quantitative real-time PCR in BV2 microglia cells pre-treated with 6 μM QUE, 50 μM NAR or 100 μM RAN for 6 h and then exposed to LPS for further 15 h. The mRNA expression values were normalized to those of 18S ribosomal and β-actin RNA, used as internal controls, and are displayed as fold change normalized to the LPS-treated sample without any pre-treatment. Data shown are the mean ± SE from n = 6–8 experiments each performed in triplicate. Asterisks denote significance; **p ≤ 0.01, ***p ≤ 0.001 and ****p ≤ 0.0001 by paired Student’s t-test. (**B**) Extracts of BV2 cells untreated (CTRL) or treated with LPS or pre-treated with 6 μM QUE, 50 μM NAR or 100 μM RAN for 6 h and then administered with LPS for further 15 h were assayed for iNOS, IL-1β and IL-6 protein levels by Western blot analysis. Density of immunoreactive bands was calculated using the Image Lab software from Bio-Rad and normalized for β-actin used as loading control. Blots cropped from different parts of the same gel are grouped and clearly delineated. Blots of the same gel are also showed in Fig. 5 therefore sharing the same loading control. Each value indicates the mean ± SE (reported as fold change normalized to the LPS-treated sample without any pre-treatment) of the densitometric analysis on three independent experiments. **p ≤ 0.01, ***p ≤ 0.00 and ****p ≤ 0.0001 by two-way repeated measures ANOVA followed by Dunnett’s multiple comparisons test, making comparison versus LPS.

**Figure 2:**
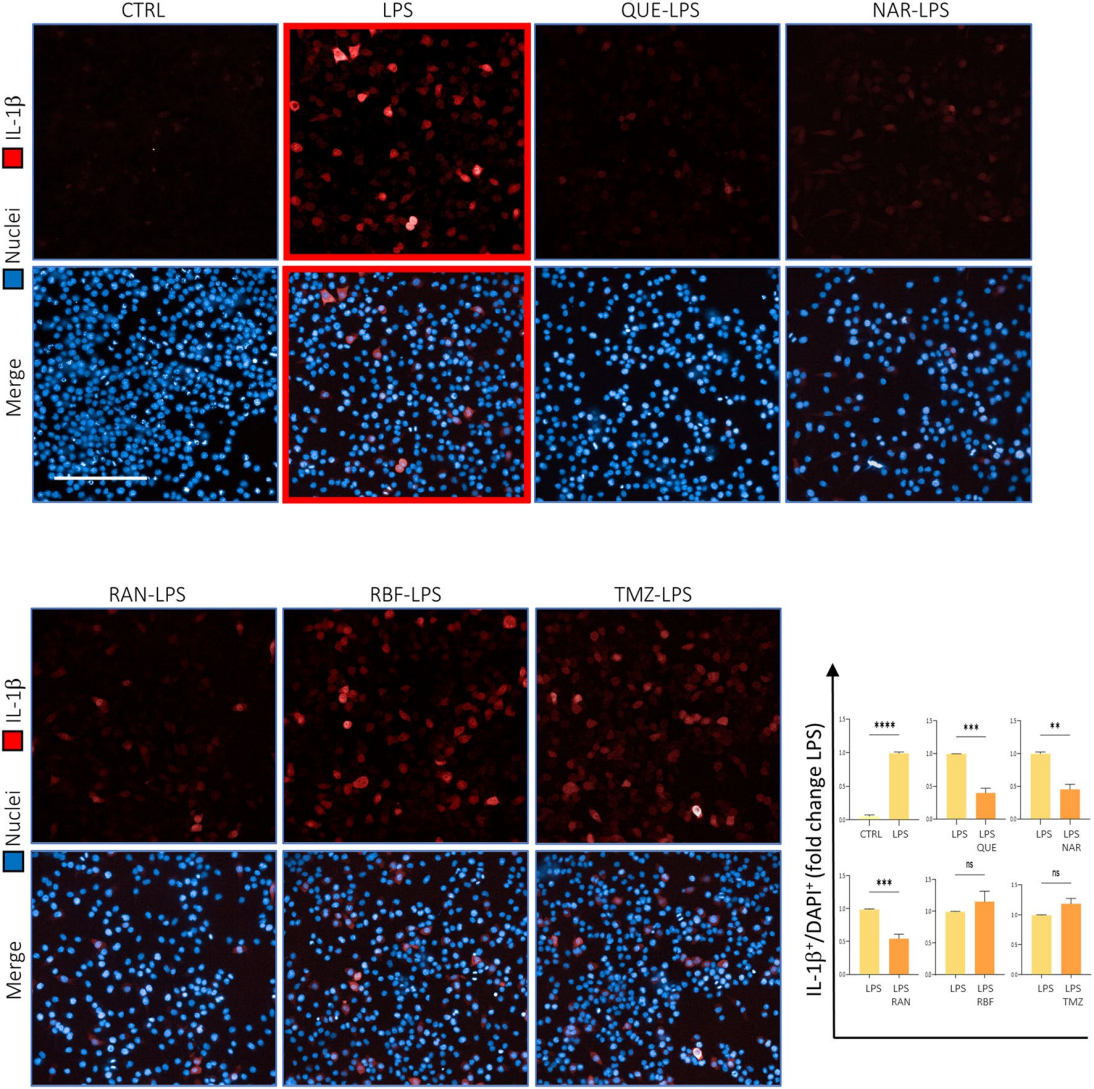
QUE, NAR and RAN reduce the number of cells expressing IL-1β. BV2 cells were untreated (CTRL) or treated with LPS or pre-treated with 6 μM QUE, 50 μM NAR, 100 μM RAN, 100 μM TMZ or 10 μM RBF for 6 h and then administered with LPS for further 15 h before fixation and immunostaining for IL-1β (red). Counterstaining with DAPI was used to visualize all nuclei (blue). The graph represents the quantification of IL-1-positive cells over the number of total nuclei in each area counted by using ImageJ software. For each condition, the number of IL-1β-positive cells over the number of total nuclei in that area were counted by using the ImageJ software. Data are the means ± SE from 3–5 independent experiments and are expressed as fold change normalized to the LPS-treated sample without any pre-treatment. **p ≤ 0.01, ***p ≤ 0.001 and ****p ≤ 0.0001 by paired Student’s test. Scale bar: 100 μm.

In order to evaluate the anti-inflammatory effect of molecules acting on metabolic pathways and specifically TMZ, RAN, RBF, IDB, QUE and NAR, we pre-treated BV2 cells with these molecules for 6 h before the endotoxin lipopolysaccharide (LPS) (100 ng/mL) administration, which lasted for further 15 h. LPS activates Toll-like receptors and stimulates signaling pathways leading to inflammatory cytokine up-regulation, as demonstrated by the overexpression pro-inflammatory molecules such as interleukin-1β (IL-1β) and -6 (IL-6), the chemokine C–C motif ligand 2 (CCL2), and the inducible NO synthase (iNOS) compared to untreated control (Supplementary Fig. S1). Thus, we tested the ability of TMZ, RAN, RBF, IDB, QUE and NAR to reduce the expression of these genes. As observed in Fig. 1a, the pre-treatment with 6 μM QUE, 50 μM NAR and 100 μM RAN had a significant effect in reducing the expression of IL-1β, IL-6, CCL2 and iNOS. In contrast, 10 μM QUE, 50 μM RAN as well as TMZ (50 μM and 100 μM) and RBF (2 μM and 10 μM) had no effect on these gene expression. Interestingly, we observed that the Q10 analogue IDB (3 μM and 5 μM) was toxic to BV2 cells, thus inducing their detachment from the dish. To confirm these data, we evaluated the protein levels of some pro-inflammatory markers by performing Western Blot analysis of IL-1β, and iNOS expression. We observed reduced levels of these proteins on BV2 cells pre-treated with QUE, NAR or RAN before LPS administration compared to not pre-treated cells (Fig. 1b). In addition, by using an allophycocyanin (APC)-conjugated anti-IL-1β antibody, we performed an immunofluorescence analysis on BV2 cells pre-treated or not with QUE, NAR, RAN, TMZ and RBF and then exposed to LPS. Cells were then analyzed by high-content confocal assay by using Operetta CLS as shown in Fig. 2. This analysis showed that IL-1β-expressing cells -absent in control samples- increase by ~ 96% upon LPS administration; however, pre-treatment with QUE, NAR or RAN reduced the number of IL-1β-expressing cells by ~ 68%, ~ 60% and ~ 50%, respectively. RBF and TMZ treatments did not show this anti-inflammatory effect (Fig. 2).

### Quercetin, Naringenin and Ranolazine anti-inflammatory effect on primary microglia cells and on zebrafish

**Figure 3:**
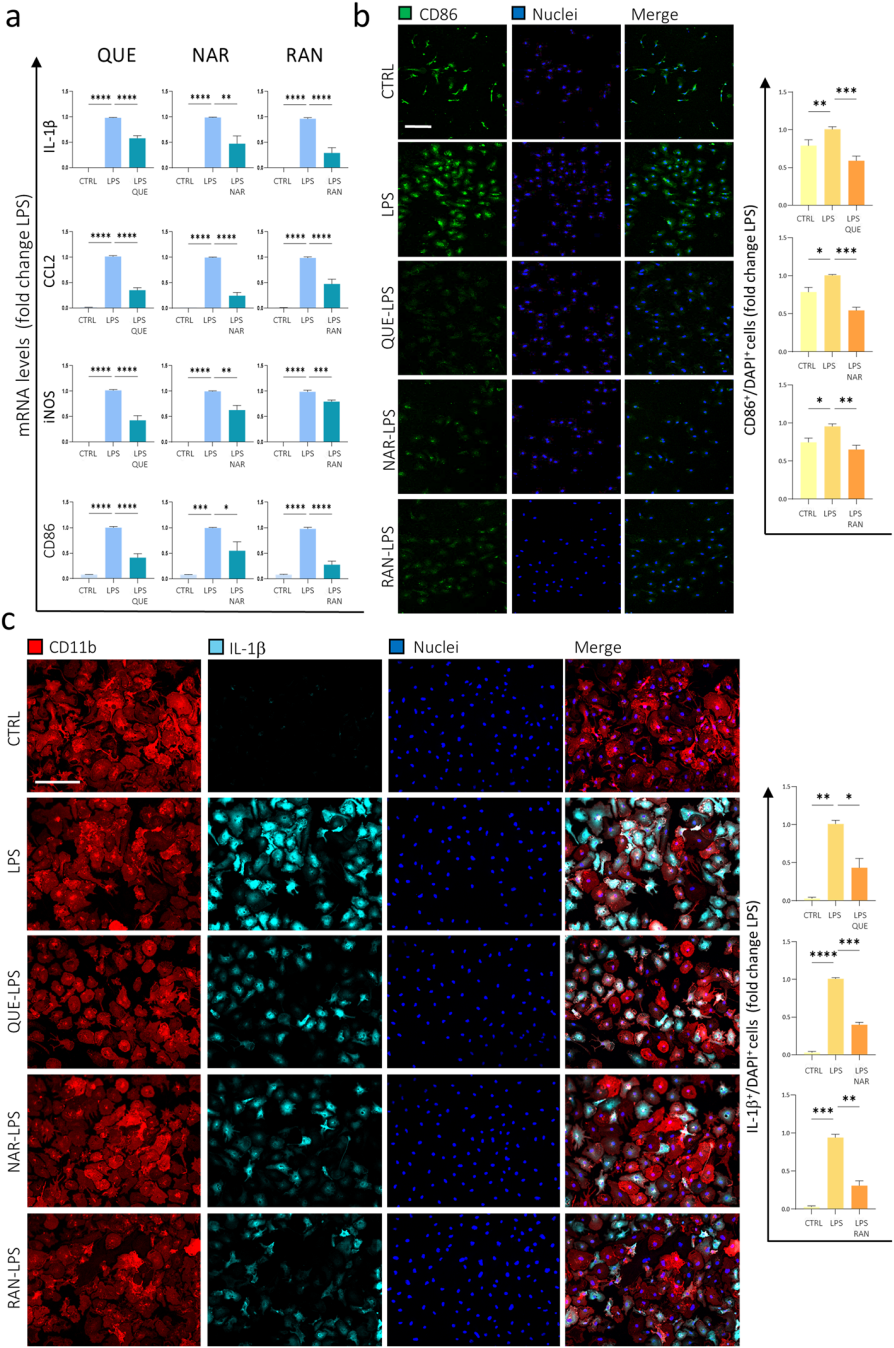
Anti-inflammatory effect of QUE, NAR and RAN on primary microglia cells. (**A**) The mRNA levels of IL-1β, CCL2, iNOS and CD86 were evaluated by qPCR in murine primary microglia cells pre-treated with 6 μM QUE, 50 μM NAR or 100 μM RAN for 9 h and then administered with LPS for further 12 h. The mRNA expression values were normalized to those of 18S ribosomal and β-actin RNAs, used as internal controls, and are displayed as fold change normalized to the LPS-treated sample without any pre-treatment (LPS). Data shown are the mean ± SE from n = 6 experiments each performed in triplicate. *p ≤ 0.05, **p ≤ 0.01, ***p ≤ 0.001 and ****p ≤ 0.0001 by one-way repeated measures ANOVA making comparison versus LPS. (**B**) Murine primary microglia cells treated as in (**A**) were fixed and immunostained for CD86 protein (green). Counterstaining with DAPI was used to visualize all nuclei (blue). The graph represents the quantification by ImageJ software of CD86 fluorescence into manually selected regions of interest (ROI) and normalized by the number of nuclei present into those areas. Data are the means ± SE from 3 experiments and are displayed as fold change normalized to the LPS-treated sample without any pre-treatment. *p ≤ 0.05, **p ≤ 0.01 and ***p ≤ 0.001 by two-way repeated measures ANOVA followed by Dunnett’s multiple comparisons test, making comparison versus LPS. Scale bar: 50 μm. (**C**) Murine primary microglia cells treated as in (**A**) were fixed and immunostained for CD11b (red) and IL-1β (cyan). Counterstaining with DAPI was used to visualize all nuclei (blue). The graph represents the quantification by ImageJ software of IL-1β fluorescence into manually selected ROI and normalized by the number of nuclei present into those areas. Data are the means ± SE from 3 experiments are displayed as fold change normalized to the LPS-treated sample without any pre-treatment. *p ≤ 0.05, **p ≤ 0.01, ***p ≤ 0.00 and ****p ≤ 0.0001 by two-way repeated measures ANOVA followed by Dunnett’s multiple comparisons test, making comparison versus LPS. Scale bar: 100 μm.

Based on these experiments, we selected QUE, NAR and also RAN as molecules having an anti-inflammatory effect on BV2 cells. To verify the validity of these data, we performed the same analysis on primary murine microglia cells isolated from WT mice cortices at post-natal day 0–2 (P0–P2). We pre-treated microglia cells with 6 μM QUE, 50 μM NAR or 100 μM RAN for 9 h followed by LPS treatment for further 12 h. qPCR analysis revealed that QUE, NAR or RAN pre-treatment reduces the expression of the pro-inflammatory markers IL-1β, CCL2 and iNOS (Fig. 3a). These results have also been confirmed by qPCR and immunofluorescence analysis of CD86, another pro-inflammatory microglia marker whose levels decrease in QUE, NAR or RAN pre-treated cells compared to LPS-only administered cells (Fig. 3a,b). In addition, the ability of QUE, NAR or RAN to reduce the levels of IL-1β was also demonstrated by immunofluorescence (Fig. 3c). In order to assess the purity of the microglia preparation, we performed CD11b staining (astrocytes are CD11b-negative) and we found that the percentage of microglia cells ranged from 92 to 98% of the total amount of cells in the dish (Fig. 3c). Notably, we also evaluated the anti-inflammatory efficacy of QUE, NAR or RAN in vivo, by performing a tailfin injury assay in transgenic zebrafish larvae expressing GFP under the control of the *mpeg* promoter, to evidence macrophages as well as microglia in zebrafish larvae^41, 42^. Preliminary evaluation suggested that the pretreatment of RAN 100 µM can reduce the number of the macrophages recruited to the site of the tail damage at 24 h post-injury (Supplementary Fig. S2). The same result was evidenced using QUE and NAR, whose anti-inflammatory efficacy had previously been described using the same zebrafish-based would healing assay^43^.

### Quercetin, Naringenin and Ranolazine effect on anti-inflammatory marker expression

**Figure 4:**
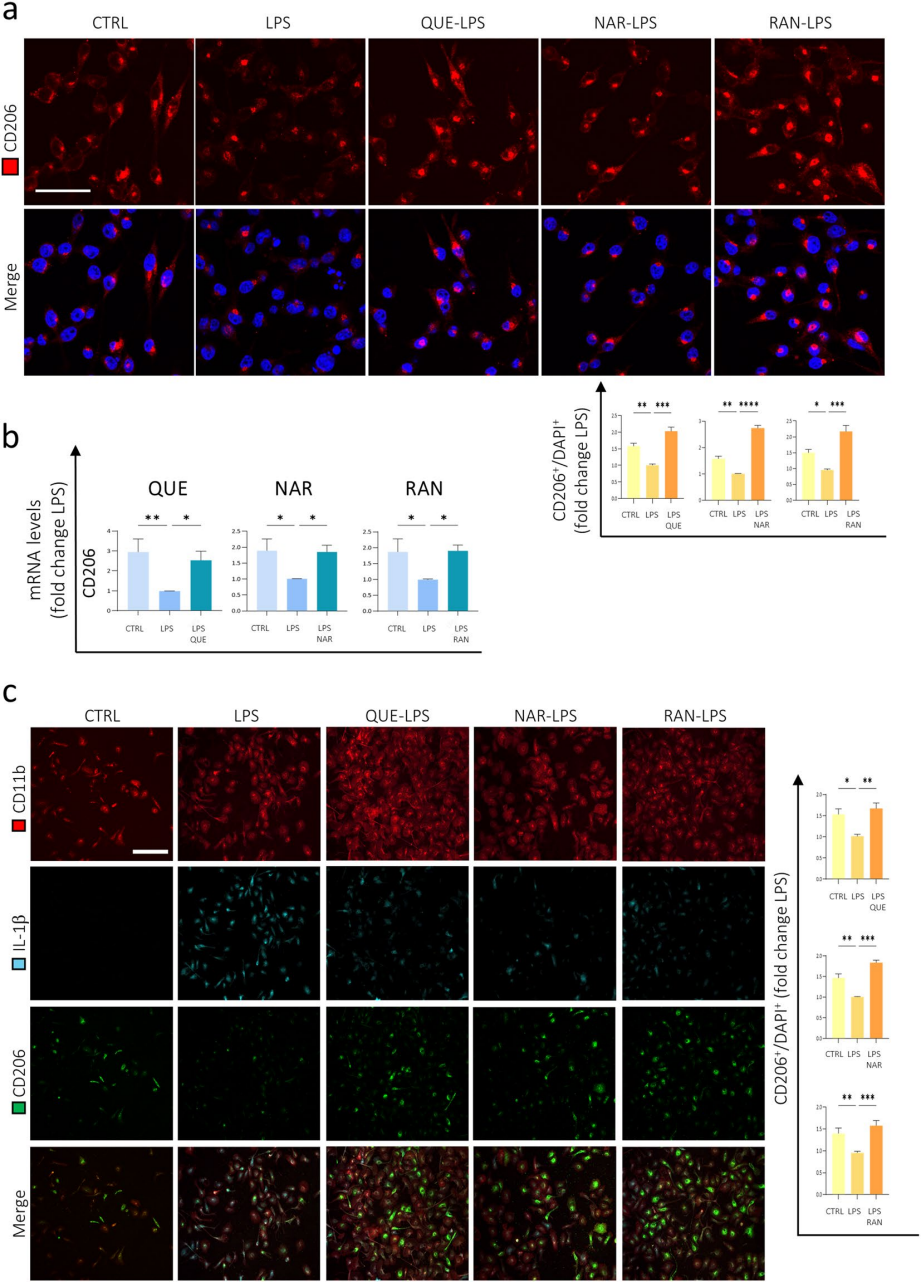
The anti-inflammatory marker CD206 is up-regulated by QUE, NAR and RAN. (**A**) BV2 cells were untreated (CTRL) or treated with LPS or pre-treated with 6 μM QUE, 50 μM NAR or 100 μM RAN for 6 h and then LPS was administered for further 15 h before fixation and immunostaining for CD206 (red). Counterstaining with Hoechst was used to visualize all nuclei (blue). The graph represents the quantification of CD206 fluorescence by ImageJ software normalized by the number of nuclei. Data are the means ± SE from 4 experiments and are expressed as fold change relative to the LPS-treated sample without any pre-treatment. *p ≤ 0.05, **p ≤ 0.01, ***p ≤ 0.001 and ****p ≤ 0.0001 by two-way repeated measures ANOVA followed by Dunnett’s multiple comparisons test, making comparison versus LPS. Scale bar: 25 μm. (**B**) The mRNA levels of CD206 were evaluated by qPCR in BV2 cells treated as in (**A**). The mRNA expression values were normalized to those of 18S ribosomal and β-actin RNAs, used as internal controls, and are displayed as fold change normalized to the LPS-treated sample without any pre-treatment (LPS). Data shown are the mean ± SE from n = 6 experiments each performed in triplicate. *p ≤ 0.05 and **p ≤ 0.01 by two-way repeated measures ANOVA followed by Dunnett’s multiple comparisons test, making comparison versus LPS. (**C**) Murine primary microglia cells were untreated (CTRL) or treated with LPS or pre-treated with 6 μM QUE, 50 μM NAR or 100 μM RAN for 9 h and then LPS was administered with for further 12 h before fixation and immunostaining for CD11b (red), IL-1β (cyan) and CD206 (green). Counterstaining with Hoechst was used to visualize all nuclei (blue). The graph represents the quantification of CD206 fluorescence into ROI by ImageJ normalized by the number of nuclei present into those areas. Data are the means ± SE from three experiments and are expressed as fold change normalized to the LPS-treated sample without any pre-treatment. *p ≤ 0.05, **p ≤ 0.01 and ***p ≤ 0.001 by two-way repeated measures ANOVA followed by Dunnett’s multiple comparisons test, making comparison versus LPS. Scale bar: 150 μm.

Once assessed the anti-inflammatory effect of QUE, NAR and RAN, we evaluated their effect on the expression of anti-inflammatory markers on microglia. We observed that QUE, NAR or RAN pre-treatment of BV2 cells increased the levels of the CD206 protein compared to BV2 cells only administered with LPS, as revealed by immunofluorescence analysis (Fig. 4a). This effect was also confirmed for QUE and RAN on CD206 gene expression (Fig. 4b). Consistently, we also found that CD206 levels increase upon QUE, NAR or RAN pre-treatment of primary murine microglia cells followed by LPS treatment, as revealed by immunofluorescence performed by using anti-CD11b, anti-CD206 and anti-IL-1β antibodies (Fig. 4c).

### Quercetin, Naringenin and Ranolazine influence the expression of mitochondrial and antioxidant genes

**Figure 5:**
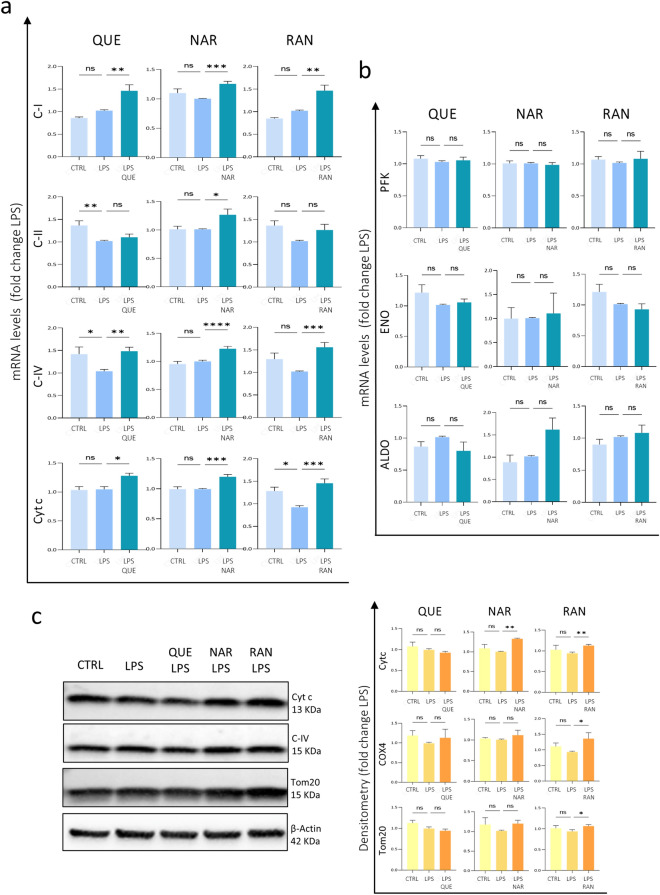
Expression of mitochondrial markers is induced by QUE, NAR and RAN. (**A**) The mRNA levels of C-I, C-II, C-IV and Cyt c were evaluated by qPCR in BV2 microglia cells pre-treated with with 6 μM QUE, 50 μM NAR or 100 μM RAN for 6 h and then administered with LPS for further 15 h. The mRNA expression values were normalized to those of 18S ribosomal and β-actin RNA, used as internal controls, and are displayed as fold change normalized to the LPS-treated sample without any pre-treatment. Data shown are the mean ± SE from n = 6–8 experiments each performed in triplicate. *p ≤ 0.05, **p ≤ 0.01, ***p ≤ 0.001 and ****p ≤ 0.0001 by two-way repeated measures ANOVA followed by Dunnett’s multiple comparisons test, making comparison versus LPS. (**B**) The mRNA levels of enolase, PFK and aldolase were evaluated by qPCR in BV2 microglia cells treated as in (A). The mRNA expression values were normalized to those of 18S ribosomal and β-actin RNA, used as internal controls, and are displayed as fold change normalized to the LPS-treated sample without any pre-treatment. Data shown are the mean ± SE from n = 3–5 experiments each performed in triplicate. (**C**) Extracts of BV2 cells treated as in (**A**) were assayed for Cyt c, C-IV and Tom20 protein levels. Density of immunoreactive bands was calculated using the Image Lab software from Bio-Rad and normalized by β-actin used as loading control. Blots cropped from different parts of the same gel are grouped and clearly delineated. Blots of the same gel are also showed in Fig. 1 therefore sharing the same loading control. Each value indicates the mean ± SE (reported as fold change normalized to the LPS-treated sample without any pre-treatment) of the densitometric analysis on three independent experiments. *p ≤ 0.05 and **p ≤ 0.01 by two-way repeated measures ANOVA followed by Dunnett’s multiple comparisons test, making comparison versus LPS.

Based on previous reports, the molecules we are analyzing are able to influence the metabolism and to reduce the oxidative stress. However, as also stated above, data in the literature on QUE, NAR or RAN activity suggest for them a wide variety of intracellular targets^20, 21, 34, 35, 37–40^. Moreover, QUE itself is easily oxidized—a modification which reduces its concentration- and the products of oxidation might either reduce QUE effect or increase it^44, 45^. Therefore, we needed to establish if the used concentrations of QUE, NAR or RAN -which we have shown to have an anti-inflammatory effect on microglia- are also able to act on metabolism and redox balance. To this aim, we analyzed the expression of some respiratory chain components, specifically complex I subunit (C-I), complex II subunit (C-II), cytochrome oxidase subunit (C-IV) and cytochrome c (Cyt c), as well as the expression of the glycolytic enzymes genes phosphofructokinase (PFK), enolase (ENO) and aldolase (ALDO) in BV2 microglia cells exposed to LPS and pretreated with QUE, NAR or RAN. We observed that QUE, NAR or RAN up-regulate most of the analyzed respiratory genes, specifically C-I, C-IV and Cyt c, whereas no influence in the expression of C-II and of the glycolytic enzymes genes was evidenced (Fig. 5a,b). Interestingly, we observed a significant or substantial increase of Cyt C, C-IV as well as Tom20 at protein level in NAR and RAN-pre-treated cells (Fig. 5c). It should be noted that the used concentration and incubation time of LPS induce neither microglial apoptosis -as demonstrated by the absence of nuclear pyknosis (Figs. 2, 3b,c and 4a)- nor Cyt c release from mitochondria. Therefore, the Cyt c quantified via Western Blot represents the amount typically found within mitochondria. In fact, we have performed immunofluorescent staining of Cyt c in BV2 cells, and Fig. S3a clearly shows that, upon LPS treatment, Cyt c is not released from mitochondria into the cytosol but fully colocalizes with the mitochondrial marker Tom20. Also, the pre-treatment with QUE, NAR or RAN followed by LPS exposure does not induce Cyt c release in the cytoplasm (Fig. S3a). Cyt c is not indeed detectable by Western blot analysis into the cytosolic fraction of untreated and treated BV2 cells (Fig. S3b).

**Figure 6:**
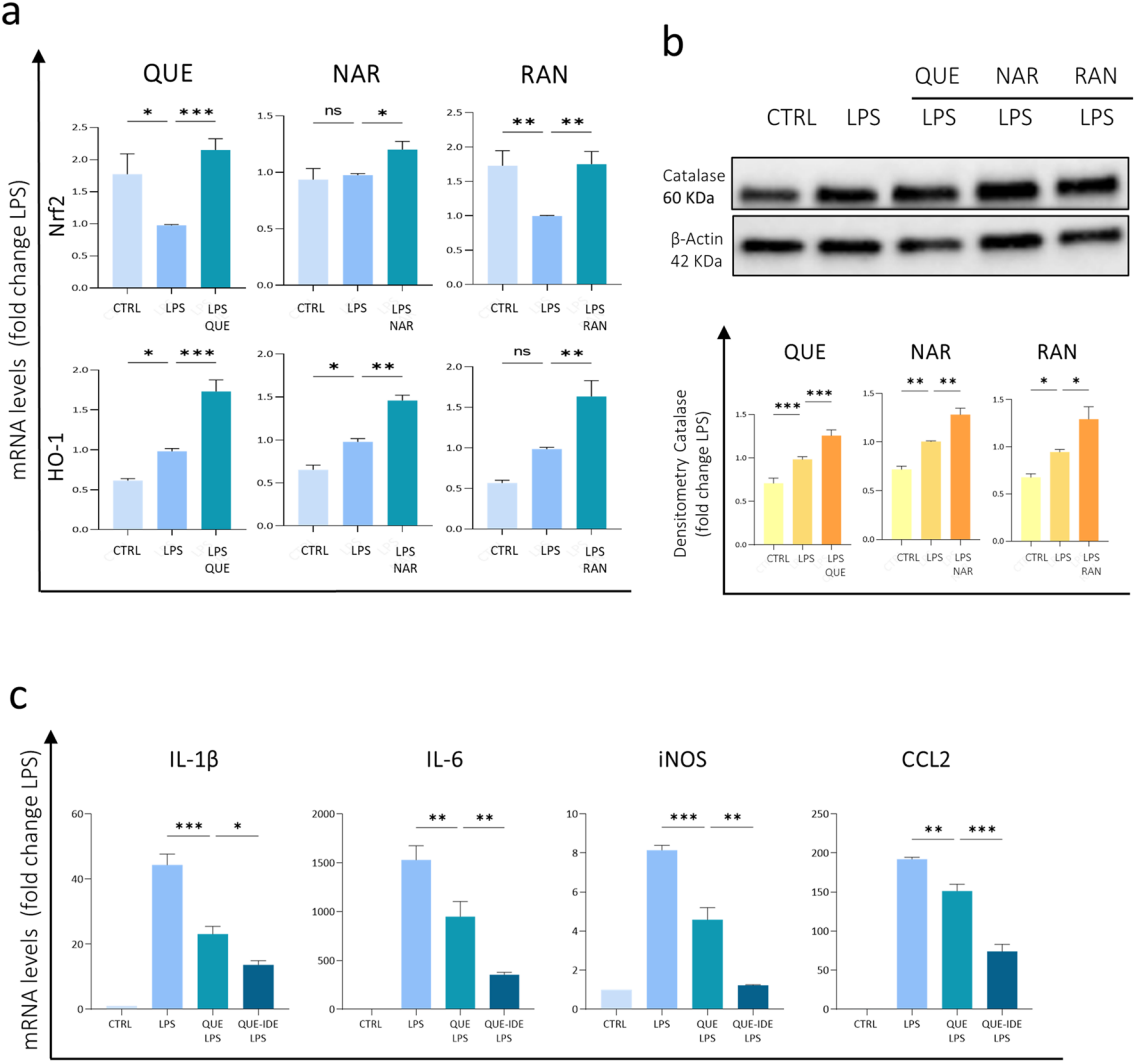
Anti-oxidant response induced by the selected molecules. (**A**) The mRNA levels of Nrf2 and HO-1 were evaluated by qPCR in BV2 microglia cells pre-treated with 6 μM QUE, 50 μM NAR or 100 μM RAN for 6 h and then administered with LPS for further 15 h. The mRNA expression values were normalized to those of 18S ribosomal and β-actin RNA, used as internal controls, and are displayed as fold change normalized to the LPS-treated sample without any pre-treatment. Data shown are the mean ± SE from n = 5–6 experiments each performed in triplicate. *p ≤ 0.05, **p ≤ 0.01 and ***p ≤ 0.001 by two-way repeated measures ANOVA followed by Dunnett’s multiple comparisons test, making comparison versus LPS. (**B**) Extracts of BV2 cells treated as in (**A**) were assayed for Catalase protein levels. Density of immunoreactive bands was calculated using the Image Lab software from Bio-Rad and normalized for β-actin used as loading control. Each value indicates the mean ± SE (reported as fold change normalized to the LPS-treated sample without any pre-treatment) of the densitometric analysis on five independent experiments. *p ≤ 0.05, **p ≤ 0.01 and ***p ≤ 0.001 by two-way repeated measures ANOVA followed by Dunnett’s multiple comparisons test, making comparison versus LPS. (**C**) The mRNA levels of IL-1β, IL-6, iNOS and CCL2 were evaluated by qPCR in BV2 microglia cells untreated (CTRL) or pre-treated with 6 μM QUE and 2 μM IDB for 6 h and then administered with LPS for further 15 h. The mRNA expression values were normalized to those of 18S ribosomal and β-actin RNA, used as internal controls, and are displayed as fold change normalized to the untreated sample. Blots cropped from different parts of the same gel are grouped and clearly delineated. Data shown are the mean ± SE from n = 3 experiments. *p ≤ 0.05, **p ≤ 0.01 and ***p ≤ 0.001 by two-way repeated measures ANOVA followed by Dunnett’s multiple comparisons test, making comparison versus QUE-LPS.

As far as the ability of QUE, NAR or RAN to affect the redox balance upon LPS-stimulation, we observed that QUE, NAR and RAN induces a significant increase of Nrf2 compared to LPS-treated sample. Interestingly, all the pretreatments also enhance the expression Nrf2-dependent gene heme oxygenase-1 (HO-1, also known as HMOX1) (Fig. 6a), an enzyme that degrades heme to generate the anti-inflammatory agent carbon monoxide CO, the anti-oxidant molecule biliverdin and free iron. We also observed an increase of the protein levels of the anti-oxidant enzyme Catalase -dismutating hydrogen peroxide into water and molecular oxygen- induced by all three molecules QUE, NAR or RAN (Fig. 6b).

### Synergistic effect of the metabolic modulators Quercetin and Idebenone

As stated above, we have found that 3 μM and 5 μM of the coenzyme Q10 analogue IDB are toxic to BV2 microglia cells. This is a controversial result if compared to data in the literature showing that IDB affects the viability of cells at higher concentrations compared to the ones we used^46, 47^*.* These are also much lower concentrations (ten to 40-fold) compared to that we used on murine and human macrophages (C. Santucci, personal communication October 10, 2022 and G. Freer, personal communication January 10, 2023). Interestingly, IDB has been approved for the mitochondrial disease Leber’s hereditary optic neuropathy (LHON), but it has been shown that about half of the patients remain unresponsive^48, 49^. There is not a clear explanation for this variable effect of IDB, but it has been suggested that this might be due to the expression ratio of two genetic variants of NAD(P)H:quinone oxidoreductase 1 (NQO1), which consistently influence the NQO1 protein levels^47, 50^*.* NQO1 is a flavoprotein catalyzing the two-electron reduction of quinones included IDB, thus avoiding the production of reactive semiquinones and favoring the quinol antioxidant activity. Once reduced by NQO1, IDB might be oxidized by complex III, thus allowing downstream electron transfer and mitochondrial respiration. Vice versa*,* if NQO1 protein levels are low, IDB is not sufficiently reduced and it might be oxidized, thus playing an opposite deleterious inhibitory effect on complex I and on respiration being not only ineffective but also detrimental. Since NQO1 is up-regulated by NRF2 in human cells and since QUE was shown to be a strong inducer of NQO1 expression^51^, we thought that it might be interesting to treat microglia cells with QUE in order to up-regulate NQO1 and, immediately after, with IDB. Notably, we found that the anti-inflammatory effect of QUE is amplified by the co-treatment with IDB (Fig. 6c), thus supporting our hypothesis of a synergistic effect played by QUE and IDB in BV2 microglia cells.

### Effect of Quercetin, Naringenin and Ranolazine-treated microglia on the photoreceptor cell line 661W

**Figure 7:**
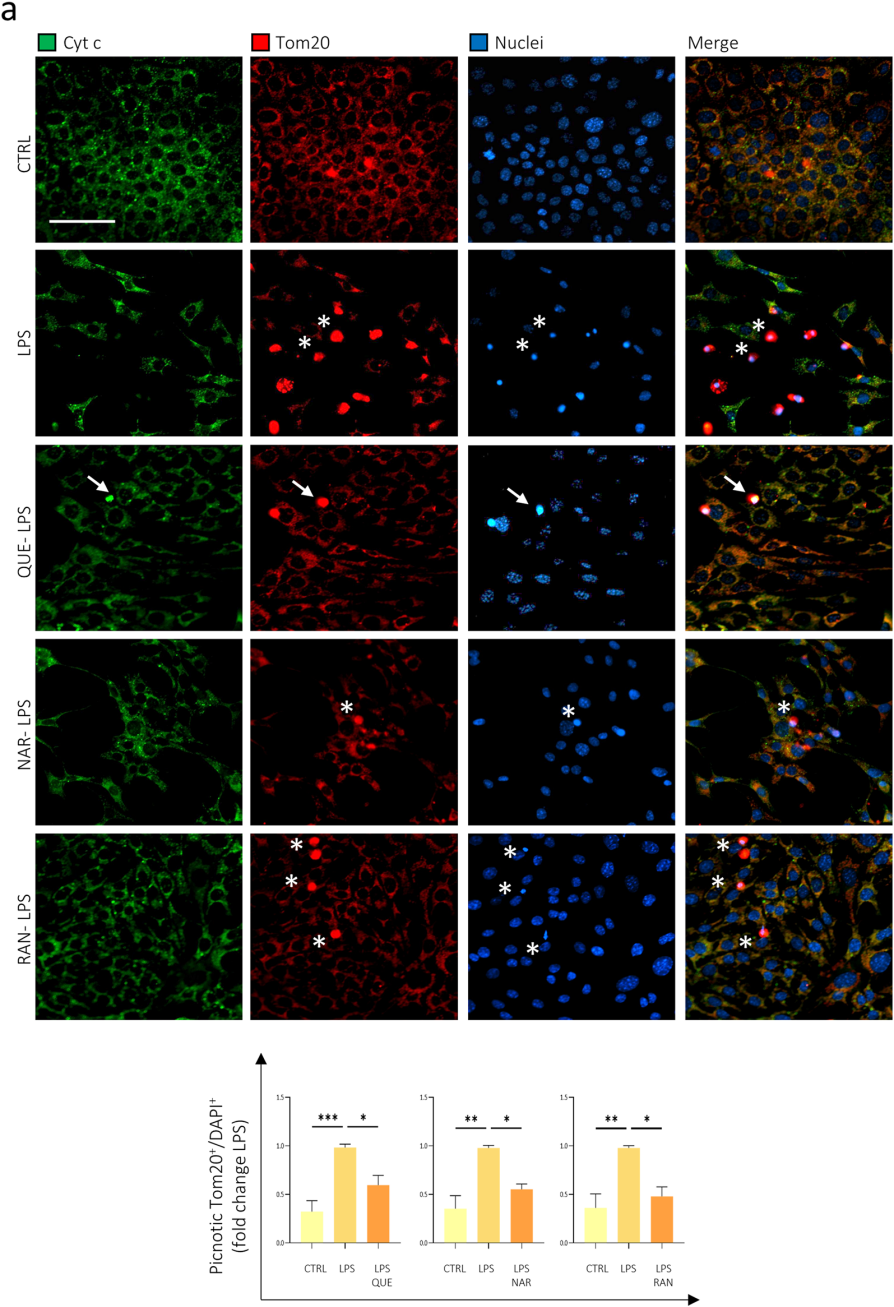

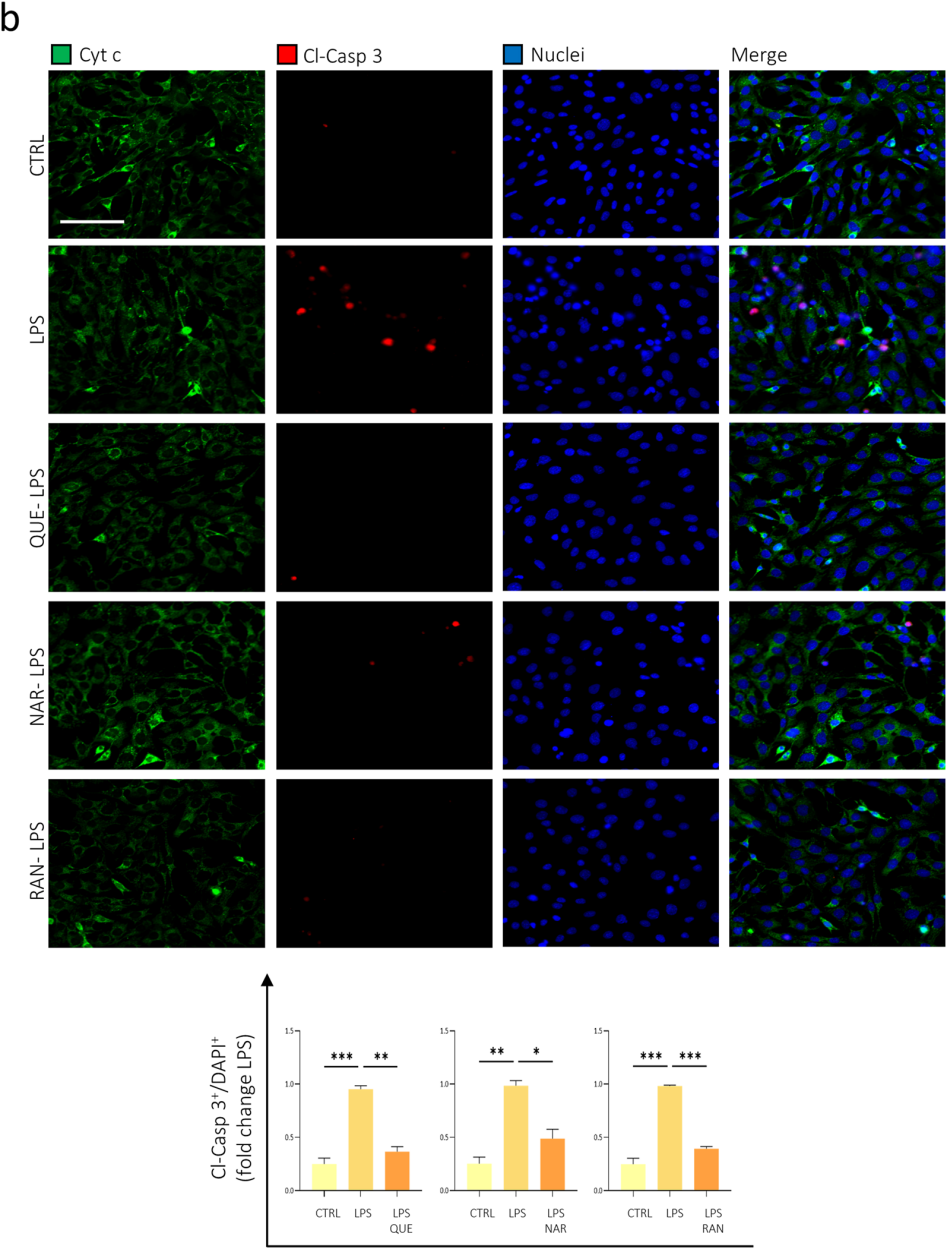
QUE, NAR and RAN pre-treatment reduces the toxicity of LPS-stimulated inflammatory microglia on the photoreceptor cell line 661W. (**A**) Differentiated photoreceptor 661W cells were incubated for 48 h with the conditioned medium of BV2 cells pre-treated with 6 μM QUE, 50 μM NAR or 100 μM RAN 6 h before LPS administration for further 12 h. 661W cells were fixed and immunostained for Cyt c (green) and Tom20 (red). Counterstaining with DAPI was used to visualize all nuclei (blue). The graph represents the quantification of apoptotic cells having pyknotic nuclei, a round shape highlighted by Tom20 staining over the number of total nuclei in each area counted by using ImageJ software. Arrows and asterisks highlight examples of cells with pyknotic nuclei maintaining or not the Cyt c labelling, respectively. Data are the means ± SE from 5 independent experiments and are displayed as fold change normalized to the sample exposed to the supernatant of LPS-treated BV2 cells. *p ≤ 0.05, **p ≤ 0.01 and ***p ≤ 0.001 by two-way repeated measures ANOVA followed by Dunnett’s multiple comparisons test, making comparison versus LPS. Scale bar: 50 μM. (**B**) Cells treated as in (**A**) were immunostained for Cyt c (green) and Cl-Casp 3 (red). Counterstaining with DAPI was used to visualize all nuclei (blue). The graph represents the quantification of apoptotic cells positive for Cl-Casp 3 over the number of total nuclei in each area counted by using ImageJ software. Data are the means ± SE from 3 independent experiments and are displayed as fold change normalized to the sample exposed to the supernatant of LPS-treated BV2 cells. *p ≤ 0.05, **p ≤ 0.01 and ***p ≤ 0.001 by two-way repeated measures ANOVA followed by Dunnett’s multiple comparisons test, making comparison versus LPS. Scale bar: 200 μM.

The data shown so far confirmed the ability of QUE, NAR, and RAN to reduce the production of pro-inflammatory and pro-oxidant molecules by microglia. Given the relevance that exacerbated inflammation and oxidative stress has on the progression of neurodegenerative diseases, including those affecting the retina, we evaluated the effect of the secretome -conditioned medium- of LPS-activated and QUE-, NAR- or RAN- pre-treated BV2 microglia cells on the viability of 661W photoreceptor-like cell line. To this purpose, we analyzed the extent of apoptosis on 661W neuronal cells incubated with the conditioned medium from LPS-activated BV2 cells with and without pre-treatment with QUE, NAR or RAN. In healthy cells, Cyt c and Tom 20 colocalize into mitochondria (see Supplementary Fig. S4; yellow arrowheads). In the first stage of apoptosis, the Cyt c is released from mitochondria; however, since the cell becomes compact, it is not possible to precisely establish the cellular localization of Cyt c and Tom20 which seem to colocalize (see Supplementary Fig. S4; arrows). At a later stage of apoptosis, the Cyt c staining disappears because, into the cytosol, it is degraded by the proteasome, whereas Tom20 is still retained into mitochondria and can be visualized (Fig S4; asterisks). We counted the dead cells having pycnotic nuclei, a round shape highlighted by the mitochondrial marker Tom20 staining which appears very intense due to the compaction of the cell, and both showing Cyt c positivity (Fig. 7a and Supplementary Fig. S4, arrows) or being Cyt c negative -due to Cyt c degradation in a later step of the apoptotic process^52^- (Fig. 7a and Supplementary Fig. S3, asterisks). We observed that the conditioned medium of LPS-stimulated microglia cells and pre-treated with QUE, NAR or RAN counteracted the reduced viability of 661W neuronal cells induced by the exposure to supernatant derived from LPS-treated microglia (Fig. 7a).

To strengthen these data, we also measured apoptosis by evaluating the number of cleaved Caspase 3 (Cl-Casp 3)-positive cells and, in accordance with the previous experiment (Fig. 6), we observed that 661W cells incubated with pre-treated QUE-, NAR- or RAN-conditioned medium were protected from apoptosis (Fig. 7b). These experiments demonstrate that QUE, NAR and RAN, by reducing the pro-inflammatory activation of microglia cells, are effective in protecting from microglial-induced neurotoxicity, in vitro. These data are consistent with the ability of QUE and NAR to reduce the neurodegeneration in two different animal models of retinitis pigmentosa, thus preserving the visual function of these animals^53, 54^*.*

## Discussion

Neurodegenerative diseases share common mechanisms including oxidative stress and neuroinflammation, and combined therapies with antioxidant, anti-inflammatory (as corticosteroids) and neurotrophic factors are able to slow down the progression of the retinal degenerative process also providing an adequate healthy environment which might increase the success of new gene- and cell-based therapies^55, 56^.

By these experiments we demonstrated, for the first time to our knowledge, the anti-inflammatory effect of the FDA-approved metabolic modulator RAN on both BV2 and primary microglia cells. RAN also reduces macrophages recruitment induced by tissue damage in vivo in zebrafish larvae, as previously described for QUE and NAR. The other analyzed piperazine derivative TMZ did not display, at the used concentrations and conditions, such an effect, nor did the RBF. In parallel, we confirmed the anti-inflammatory activity of both flavonoids QUE and NAR on BV2 microglia cells, and we demonstrated that NAR counteracts inflammatory activation of LPS-administered primary microglia cells, which had not been previously reported in the literature.

The anti-inflammatory effect of QUE is considered dependent on its anti-oxidant role and, in particular, to its ability to induce the anti-oxidant pathway involving Kelch-like ECH-associated protein 1 (Keap1)/Nrf2^33, 57^*.* Nrf2 is a bZIP transcription factor binding to the anti-oxidant response elements (AREs) in the promotor of many genes -including its own promoter^58^- encoding for cytoprotective and anti-oxidant proteins. In homeostatic conditions, Nrf2 levels are kept low because Keap1 and Cullin3 target Nrf2 for proteasome degradation. QUE leads to an increase of Nrf2 protein levels, which translocate into the nucleus and activates anti-oxidant genes among which HO-1. The metabolic modulator RAN has previously been proposed to have a systemic anti-inflammatory role and to counteract oxidative stress in vivo^21, 59^. Here we demonstrated that RAN has an anti-oxidant action also on microglia as revealed by Nrf2 and HO-1 over-expression. Although it has been suggested that inhibition of Na^+^ influx promoted by RAN might be the cause of reduced expression of pro-inflammatory cytokines^60^*,* here we propose that, similarly to QUE, the anti-inflammatory activity of RAN on microglia might also be associated to this anti-oxidant role.

As previously suggested for the M2-like pro-regenerative activation of macrophage cells, here we also correlated the anti-inflammatory activity of QUE, NAR and RAN to their ability to stimulate the expression of mitochondrial markers on microglia cells, whereas glycolytic genes remain unaffected. In this context, we need to underline an extreme variability of the effect of QUE at the protein levels (see also Supplementary Fig. S5), which might be explained by the considerable instability of the studied molecules^44, 45^ also consistent with ongoing research conducted by D. Puppi (personal communication March 6, 2023). In particular, QUE might exert opposite (cytotoxic or beneficial) effects depending on its concentration^61^ which, obviously, also depends on its stability.

Interestingly, we have also found that QUE and IDB co-treatment has a synergistic anti-inflammatory effect, thus supporting our hypothesis by which QUE-dependent induction of NQO1 expression might contribute reducing IDB which, in turn, promotes respiratory chain electron transfer. This metabolic shift toward the mitochondrial metabolism might enhance the anti-inflammatory activation of microglia. In addition, since flavonoids are also precursors of quinones—e.g. NAR is a direct precursor of these molecules^62^- we can speculate that an increase of quinones due to flavonoids but not associated to a parallel increase of NQO1 might be useless or noxious. In this view, the up-regulation of NQO1 triggered by QUE and NAR should be part of their antioxidant effect but should also be crucial to support their role as precursors of quinones. Being IDB and quinones NQO1-dependent, combination therapies using drugs able to safely promote NQO1 expression may enhance the neuroprotective therapeutic potential of quinones and their precursors, flavonoids. Finally, since NQO1 role is partly related to the reduction of free radical load, and since NQO1 is a flavoprotein tightly binding the FAD cofactor, also the co-treatment with RBF might potentially have an additive effect. This point deserves further investigation, however; experiments aimed at elucidating the potential synergistic effect of the studied metabolic modulators, along with their effect on neuronal 661W cells, are ongoing.

In conclusion, our results show that QUE, NAR and RAN are able to mitigate the neurotoxic effects induced by cytotoxic and pro-oxidant factors released by LPS-activated microglia. This underscores and suggests a preventive and therapeutic potential of these compounds to ameliorate neurodegenerative diseases, with particular reference to retinal neurodegeneration. However, due to their potential synergy, and due to the variability of their effects which also depends on their concentrations, they would likely be more effective if stabilized in micro- or nano-formulations and/or if administered in combination therapies. Notably, the anti-inflammatory effect of the metabolic modulator RAN -also able to increase cell metabolic efficiency, and to counteract oxidative stress while avoiding corticosteroid side effects- makes this drug appealing for its possible repositioning for the treatment of retinal neurodegenerative diseases. RAN is particularly interesting also considering that it has a high safety profile, has already been approved for clinical use, and it is more stable compared to the other analyzed flavonoids.

## Methods

### BV2 cell culture and treatments

Murine BV2 microglia cells (American Type Culture Collection; ATCC, Manassas, USA) were tested for contamination and then grown in DMEM (Dulbecco’s Modified Eagle’s Medium) high glucose with Glutamax, 5% Fetal Bovine Serum (FBS) and 1% penicillin/streptomycin at 37 °C in a 95% O_2_ and 5% CO_2_ humidified chamber. All reagents for media preparation were from Thermo Fisher Scientific (Monza, Italy). Cells were seeded at 1.2 * 10^4^/cm^2^, the day after were pre-treated with with 6 μM QUE (Merck), 50 μM NAR (Merck) or 100 μM RAN (Selleckchem) for 6 h and then LPS (Merck) was added for further 15 h.

Photoreceptor cell line 661W cells were grown in DMEM with 10% FBS. To induce differentiation, cells were seeded and after 24 h exposed to the medium without serum for 4 h. After differentiation, photoreceptor 661W cells were incubated with 3% FBS medium until the day after when they were incubated for 48 h with the conditioned medium of BV2 cells pre-treated with 6 μM QUE, 50 μM NAR or 100 μM RAN 6 h before LPS administration for further 12 h.

### Primary microglia cell isolation and treatments

Primary cultures of microglia were obtained from CD-1 mice brain at P0–P1. After removal of meninges, the explanted cortices were transferred to a Petri dish in cold HBSS (Hank's Balanced Salt Solution) without Ca^2+^ and Mg^2+^ containing 10 mM HEPES (Gibco). Subsequently, the isolated cortices were cut into small pieces and digested with 0.25% trypsin and 0.01% DNaseI. After dissociation and passage through 70-mm filters, cells were resuspended in high glucose DMEM-F12 supplemented with Glutamax, 10% FBS and 1% penicillin/streptomycin and the cells obtained from 1 cortex were seeded on a surface of 45 cm^2^, on 24 well plates for RNA extraction and on coverslip placed on 35 mm dishes for immunofluorescence. After 15 days, the astrocytes were removed by incubating the cells with Trypsin diluted 1:1 with DMEM for 45 min at 37 °C; the removal of astrocytes left 99% pure microglial cells adherent to the dish^63^. After 24–48 h, primary microglial cells were pre-treated with the studied molecules for 9 h after which 100 ng/mL LPS was added for further 12 h.

### Fluorescence microscopy

BV2 cells were seeded at 1.2*10^4^/cm^2^ on coverslips placed on 35 mm dishes, whereas primary microglia cells were obtained as above reported. The day after, cells were treated as previously reported and, at the end of the treatment, were fixed in 4% paraformaldehyde (PFA) or ice-cold acetone. The permeabilization step (only for PFA-fixed cells) was performed with 0.4% Triton-X-100 in PBS and the blocking step with 2% horse serum in PBS. After the blocking step, cells were incubated overnight with the primary antibody at 4 °C (Table 1). Samples were then washed and incubated for 1 h with a secondary antibody (1:500; Thermo Fisher Scientific) at room temperature. Nuclei were counterstained with 4’,6 diamidino-2-phenylindole (DAPI, Thermo Fisher Scientific), washed with PBS and the coverslip was mounted on a slide in Aqueous Mounting solution (Sigma-Aldrich, USA). The images were acquired with the confocal microscope Nikon-A1.

**Table 1:** List of primary antibodies.

Antibody	Host	Company	Code
Anti Cyt c	Mouse	BD Biosciences	556432
Anti Tom20	Rabbit	Santa Cruz Biotechnology	SC-11415
Anti Cl-Casp3	Rabbit	Sigma-Aldrich	ab3623
Anti CD206	Goat	RD Systems	AF2536
Anti IL-1β	Human	Milteny	130-109-042
Anti CD86	Rat	BD Biosciences	550542
Anti CD11b	Mouse	Milteny	130-113-806
Anti C-IV	Rabbit	GeneTex	GXGTX114330
Anti β-Actin	Mouse	Sigma-Aldrich	A5228
Anti iNOS	Mouse	RD Systems	MAB9502
Anti MnSod	Rabbit	Enzo	adi-sod-110f
Anti Catalase	Mouse	Sigma-Aldrich	C0979
Anti GAPDH	Mouse	Sigma-Aldrich	G8796

### High-content confocal assays

BV2 cells were seeded in 96-CellCarrierUltra plates (Perkin Elmer, Hamburg, Germany), then treated as described above. Cells were fixed using 3.7% formaldehyde/PBS solution and stained with antibody against IL-1β and DAPI (1 µg/mL). Images were acquired using Operetta CLS high-content imaging device (PerkinElmer, Hamburg, Germany) and analyzed with Harmony 4.6 software (PerkinElmer Hamburg, Germany). To investigate the cytoplasm intensity of IL-1β, we counted an average of 45 fields and ~ 5000/6000 cells/well. Images were analyzed with the following building blocks: Find Nuclei > Find Cytoplasm > Calculate intensity properties (IL-1β 647) > Select population: IL-1β + cells > Calculate number of IL-1β + BV2 cells out of total events. Images were acquired with 40 × water magnification as previously described^64^.

### Quantitative real-time PCR (qPCR)

The RNA isolation from BV2 and primary microglia cells seeded on 12-well plates (4.4 * 10^4^/well) and 24-well plates (2.2 * 10^4^/well), respectively, and treated as above reported was performed by using NucleoSpin RNA Plus (MACHEREY–NAGEL) following the manufacturer’s instructions. The cDNA was synthesized by using 1–3 mg RNA and the GoScript Reverse Transcription System (Promega) with Random Primers. Comparative qPCR was performed with the SensiMix SYBR No-ROX (Meridian Bioscience) by using the QuantStudio 3 (Applied Biosystem by Thermo Fisher Scientific). Data were normalized to housekeeping genes β-actin and TBP. Resulting data were analysed by using the 2^−ΔΔCT^ method. All reactions were performed in duplicate.

The following primers were used:

**Table:** 

Target	Forward sequence	Reverse Sequence
IL-1β	5′-GAC CTT CCA GGA TGA GGA CA-3′	5′-TCC ATT GAG GTG GAG AGC TT-3′
CCL2	5′-AGG TCC CTG TCA TGC TTC T-3′	5′-CTC CAG CCT ACT CAT TGG GA-3′
IL-6	5′-TCC TCT CTG CAA GAG ACT CC-3′	5′- TTG TGA AGT AGG GAA GGC CG-3′
CD206	5′-ATC CAC TCT ATC CAC CTT CA -3′	5′-TGC TTG TTC ATA TCT GTC TTC A -3′
C-IV	5′CAG AAG GGA CTG GAC CCA TA-3′	5′-ATA ACA CAG GGG CTC AGT GG-3′
Cyt c	5′-GCC CGG AAC GAA TTA AAA AT-3′	5′-CCA GGT GAT GCC TTT GTT CT-3′
iNOS	5′GGG TGG CCT CGA ATT CCC AG-3′	5′-CCA TCC TTC GGC CCA CTT CC-3′
ALDO	5′-TTA GTC CTT TCG CCT ACC CA-3′	5′-GCG ATG TCA GAC AGC TCC TT-3′
HO-1	5′-CCC ACC AAG TTC AAA CAG CTC-3′	5′-AGG AAG GCG GTC TTA GCC TC-3′
CD86	5′-ATC AAG GAC ATG GGC TCG TA-3′	5′-TTA GGT TTC GGG TGA CCT TG-3′
Nrf2	5′-AGC AGG ACA TGG AGC AAG TT-3′	5′-TTC TTT TTC CAG CGA GGA GA-3′
ENO	5′-CAC CCT CTT TCC TTG CTT TG-3′	5′-AGA TCG ACC TCA ACA GTG GG-3′
C-I	5′-TTG GGA ACA ACA GGA AGA GG-3′	5′-TTC CCA CTG CAT CCA TTA CA-3′
C-II	5′-CTG GTG GAA CGG AGA CAA GT-3′	5′-GTT AAG CCA ATG CTC GCT TC-3′
18S	5′-CCC TGC CCT TTG TAC ACA CC-3′	5′-CGA TCC GAG GGC CTC ACT A-3′
β-Actin	5′-TTG AAT CCT GTG GCA TCC ATG AAA C-3′	5′-TTA AAC GCA GCT CAG TAA CA-3′
TBP	5′-ACG CTT CAC CAA TGA CTC CTA TG-3′	5′-TGA CTG CAG CAA ATC GCT TTG G-3′

### Protein isolation and Western blotting

BV2 and primary microglia cells seeded on 35 mm dishes (1.1 * 10^5^/cm^2^) were washed twice in PBS and lysed at 4 °C in RIPA lysis buffer (50 mM Tris/HCl, pH 8, 150 mM NaCl, 0.5% sodium-deoxycholate, 1 mM EDTA, 0.1% NP40, 0.1% SDS) supplemented with Protease Inhibitor Cocktail (Sigma-Aldrich). A clear supernatant was obtained by centrifugation of lysates at 13,000 g for 20 min at 4 °C. Protein concentration in the supernatant was determined by Bradford protein assay (Bio-Rad).

Depending on the analyzed protein, 5–30 μg of samples were then separated by SDS-PAGE using Mini-protean TGX Gels precast gels (Bio-Rad) and proteins were transferred to nitrocellulose membranes (Bio-Rad). Membranes were blocked 1 h at RT with 5% non-fat milk in T-TBS (Tris-Buffered Saline with 0.1% Tween 20). Incubation with primary antibodies (Table 1) was performed in 2% non-fat milk in T-TBS over night at 4 °C. The next day, after washes in T-TBS the membrane was incubated with the secondary horseradish peroxidase-conjugated antibodies (Jackson Immunoresearch) in 1% non-fat milk in T-TBS (1:5000). Immunoreactive bands were visualized by adding Clarity Western ECL Substrate (BioRad) and acquired by ChemiDoc XRS (Bio-Rad). Each protein of interest was normalized for the content of a specific reference protein (β-actin). Densitometry was undertaken using Bio-Rad ImageLab software.

### Subcellular fractionation

BV2 cells were harvested in hypotonic buffer (2 mM MgCl2, 10 mM KCl, 10 mM Tris–HCl, pH 7.6) and incubated for 20 min on ice. In all, 1 V of 2X Mitobuffer (450 mM sucrose, 10 mM Tris–HCl, pH 7.4, 400 µM EDTA, pH 7.4, 2 µM DTT) supplemented with protease inhibitor cocktail (SIGMA) was added and the cell suspension was homogenized for 100 strokes with a Dounce homogenizer. Samples were centrifuged at 900 g for 5 min at 4 °C to eliminate cell nuclei and unbroken cells. The resulting supernatant was centrifuged at 16,000 g for 30 min at 4 °C to recover the heavy-membrane pellet enriched for mitochondria, and the resulting supernatant was stored as the cytosolic fraction.

### Zebrafish tailfin injury assay

Zebrafish larvae were obtained by natural mating of Tg (*mpeg1*:GFP) fish, where the expression of GFP in macrophages is driven by the *mpeg1* promoter, and maintained in the incubator at 28 °C in E3 media, according to the ZFIN procedures. Zebrafish larvae at 3 days post-fertilization (dpf) were pre-treated with 30 μM naringenin, 30 μM quercetin, 30, 100 or 150 μM ranolazine in E3 media and incubated at 28 °C. Controls were pre-treated with 1% DMSO diluted in E3 media. After 6 h, treatment solutions were removed and stored, and larvae were washed in E3 media and were anesthetized in 0.02% tricaine solution. To induce an inflammatory condition, tailfin transection was performed using a 10-blade sterile scalpel^65^. To evaluate the effect of the anti-inflammatory compounds on macrophage recruitment, larvae were imaged at 0-, 5- and 24-h post-injury (hpi) with a Nikon SMZ18 stereomicroscope in bright-field and FITC channels to detect the presence of GPF-positive macrophages. After imaging, larvae were immersed in the treatment solutions (one larva/well) and incubated at 28 °C. Data analysis was performed for each larva at each time point using ImageJ software by counting the number of GFP-positive cells within a defined region of interest (ROI).

### Ethical approval

All animal procedures were conducted in accordance with European Guidelines for the use of animals in research (2010/63/EU), approved by the local ethics committee (Italian Ministry of Health-Direzione Generale della Sanità Animale e dei Farmaci Veterinari; Protocol Number 186/2022-PR) based on the requirements of Italian laws (the National Ethical Guidelines by Italian Ministry of Health; D.lgs. 26/2014) and conformed to ARRIVE guidelines 2.0 to improve the reporting of research involving animals. The investigators declare that the principle of the “3R” (replace, reduce and refine) was carefully fulfilled.

### Supplementary Information

Supplementary Figures.

## Data Availability

All data generated or analyzed during this study are included in this published article.
