# Supplementary material for: Anti-inflammatory reprogramming of microglia cells by metabolic modulators to counteract neurodegeneration; a new role for Ranolazine
Source: Sci Rep. 2023 Nov 17;13:20138. doi: 10.1038/s41598-023-47540-8 (PMC10656419; doi:10.1038/s41598-023-47540-8)
Supplement: Supplementary file 1 — Supplementary Figures. [file 41598_2023_47540_MOESM1_ESM.pdf]

Anti-inflammatory reprogramming of microglia cells by metabolic modulators to counteract neurodegeneration; a new role for Ranolazine

Ilaria Piano, Arianna Votta, Patrizia Colucci, Francesca Corsi, Sara Vitolo, Chiara Cerri, Dario Puppi, Michele Lai, José Fernando Maya-Vetencourt, Massimiliano Leigheb, Chiara Gabellini and Elisabetta Ferraro.

Supplementary
Figure S1

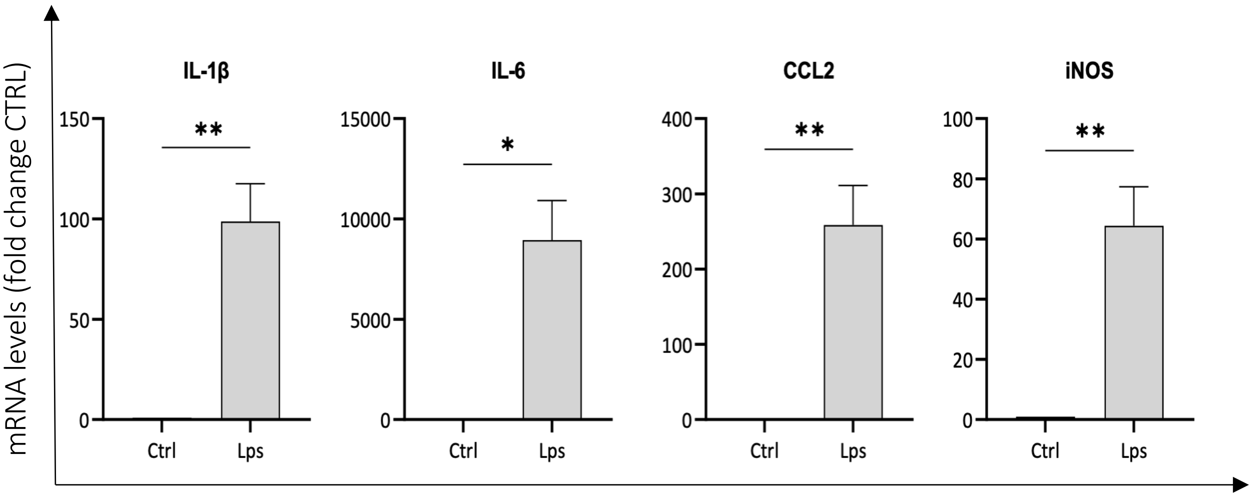

Figure S1

Pro-inflammatory activation of BV2 cells by LPS.

The mRNA levels of IL-1β, IL-6, CCL2 and iNOS were evaluated by qPCR in BV2 microglia cells administered with LPS for 15 hours. The mRNA expression values were normalized to those of 18S ribosomal and β-actin RNA, used as internal controls, and are displayed as fold change normalized to the untreated sample (CTRL). Data shown are the mean ± SE from n=6-8 experiments each performed in triplicate. Asterisks denote significance; *p ≤0.05 and **p≤0.01 by paired Student's t-test.

Supplementary Figure S2

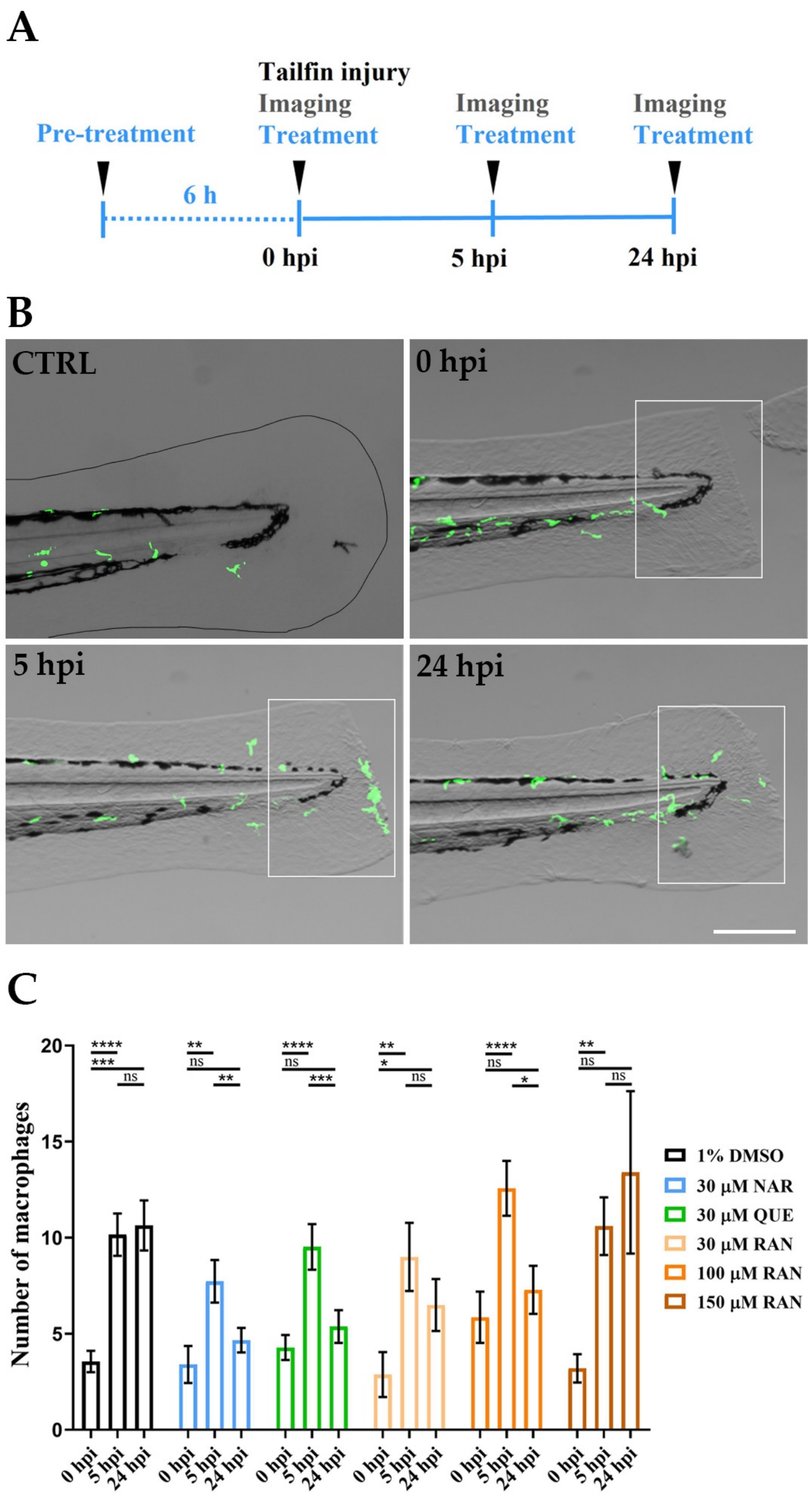

Figure S2 QUE, NAR and RAN reduce the number of macrophages recruited after injury *in vivo*. (A) Experimental timeline. Tg (*mpeg1*:GFP) zebrafish larvae at 3 days post-fertilization were pre-treated with 30 µM QUE, 30 µM NAR, 30 µM, 100 µM or 150 µM RAN. Control larvae were treated with 1% DMSO solution. Then, tailfin transection was performed, and larvae were imaged at 0, 5 and 24 hours post-injury (hpi). The number of GFP-positive macrophages was quantified in each larva over time. (B) Representative images showing an increase of macrophages recruited over time at the tailfin after transection (0, 5 and 24 hpi) compared to uncut tailfin (CTRL). Scale bar: 100 µm. (C) Evaluation of the anti-inflammatory effect of tested compounds on macrophage recruitment after tailfin injury in zebrafish larvae. The graph shows the quantification of GPF-positive cells. For each condition, the number of GPF-positive cells was counted at 0, 5 and 24 hpi by using the ImageJ software. Data are expressed as the mean ± SEM from 1-4 independent experiments. ns, $p > 0.05$, *$p \leq 0.05$, **$p \leq 0.01$, ***$p \leq 0.001$ and ****$p \leq 0.0001$ by two-way ANOVA followed by Tukey's multiple comparisons test.

Supplementary
Figure S3a

a

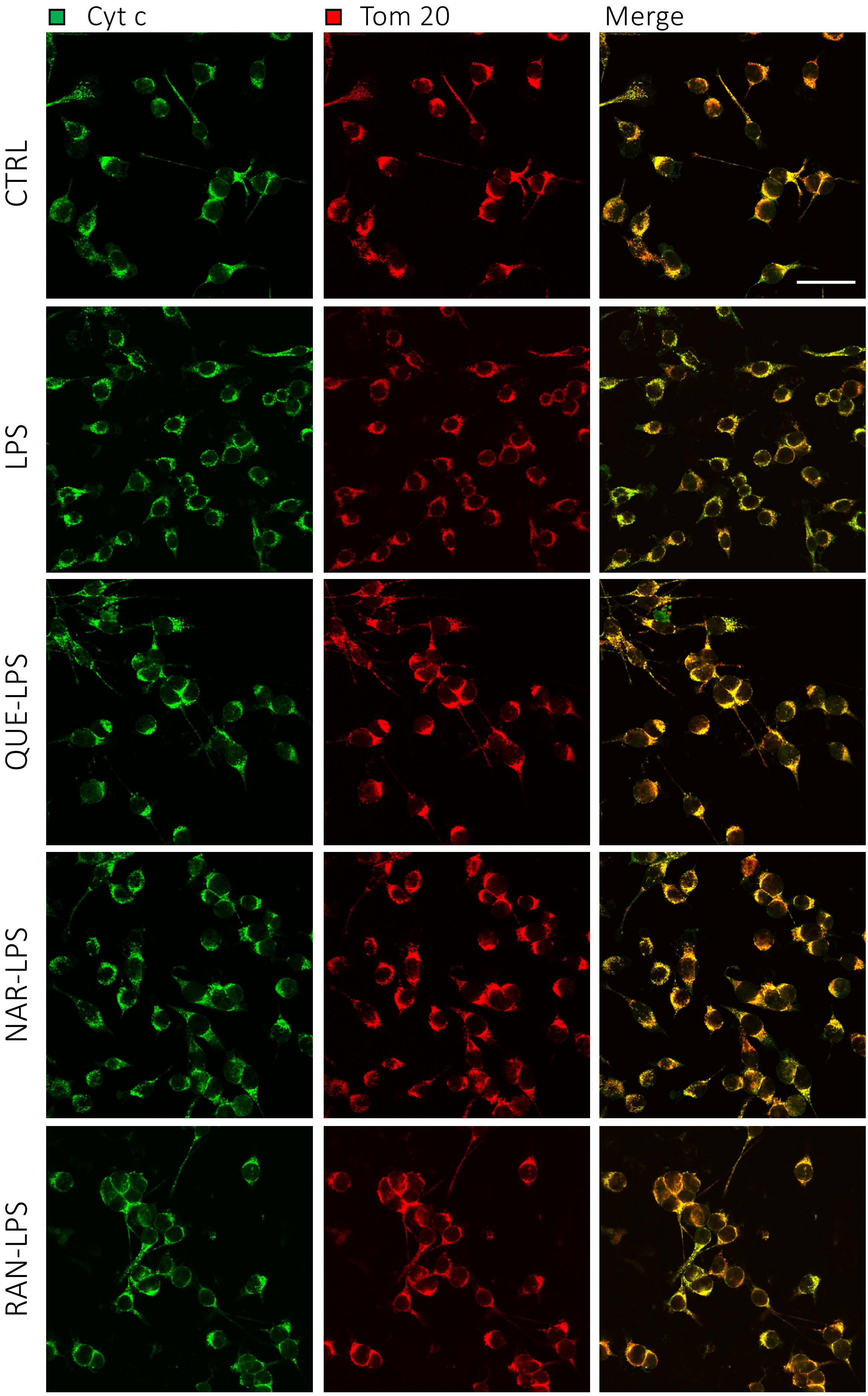

Supplementary
Figure S3b

b

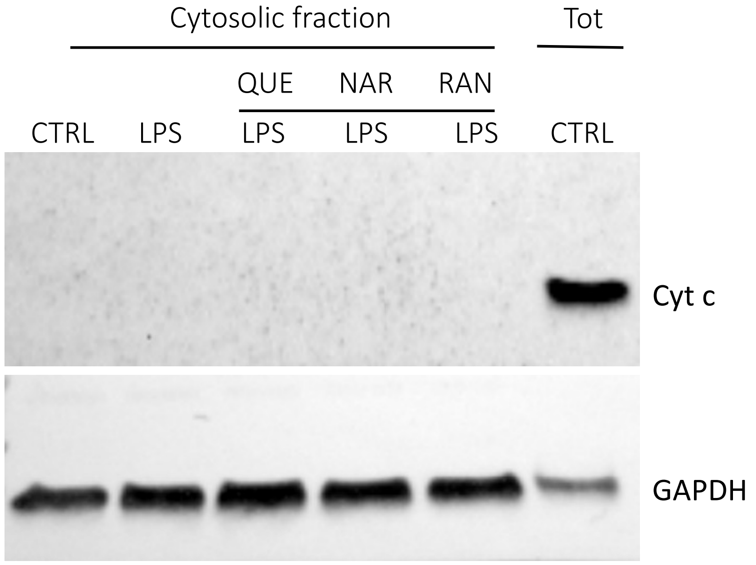

FIGURE S3. LPS does not induce BV2 cell death or Cyt c release from mitochondria. (a) BV2 cells pre-treated with 6 μM QUE, NAR or RAN for 6 hours and then administered with LPS for further 15 hours were were fixed and immunostained for Cyt c (green) and Tom20 (red). Scale bar: 50 μm. **(b)** BV2 cells pre-treated with 6 μM QUE for 6 hours and then administered with LPS for further 15 hours were fractionated in order to obtain the cytosolic fraction which, along with the same amount of whole cell lysate (Tot) obtained from untreated BV2 and used as positive control for Cyt c presence, were assayed for Cyt c and for GAPDH used as normalizer.

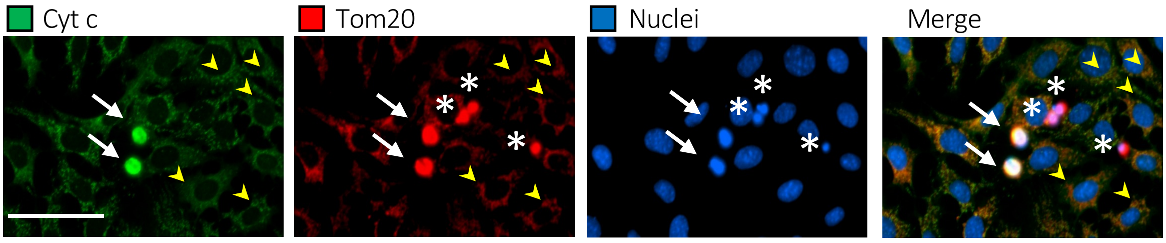

Figure S4. Examples of apoptotic cells at various stages in differentiated photoreceptor 661W cells exposed to the supernatant of BV2 microglial cells treated with LPS. Differentiated 661W cells were incubated for 48 h with the conditioned medium of BV2 cells treated with LPS for 18 hours. Cells were fixed and immunostained for Cyt c (green) and Tom20 (red). Counterstaining with DAPI was used to visualize all nuclei (blue). Apoptotic cells are distinguished by the presence pyknotic nuclei and by a round shape highlighted by Tom20 staining. Examples of cells with Cyt C mitochondrial localization (yellow arrowheads) with pyknotic nuclei and maintaining (arrows) or not (asterisks) the Cyt c labelling are reported. Scale bar: 50 µm.

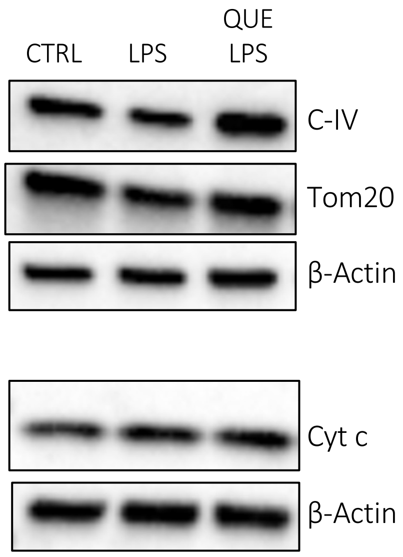

Figure S5. Variability of modulation of mitochondrial markers levels upon QUE treatment compared to Fig 5C. Extracts of BV2 cells pre-treated with 6 μM QUE for 6 hours and then administered with LPS for further 15 hours were assayed for Cyt c, C-IV and Tom20 protein levels, as reported in Figure 5C.

Original WB DATA

1A
Example of how filters are cut before antibody incubation

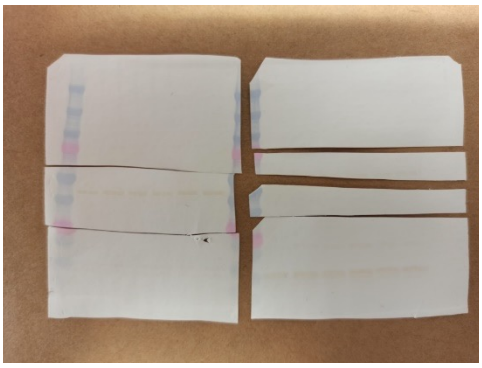

1B
Figure 1B and 5C
Original blots

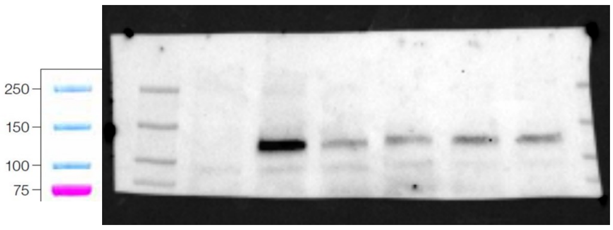

Fig 1b iNOS

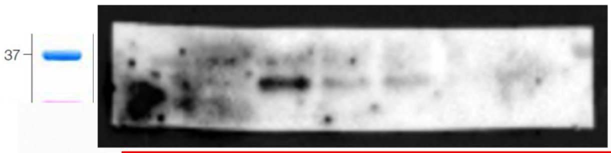

Fig 1b IL-1β

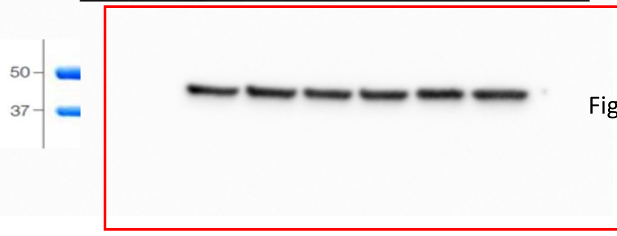

Fig 5C β-Actin

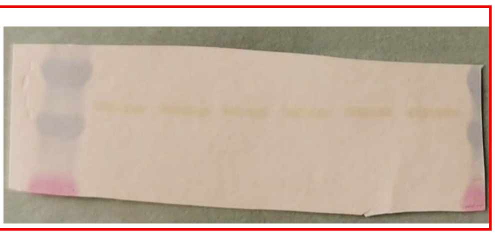

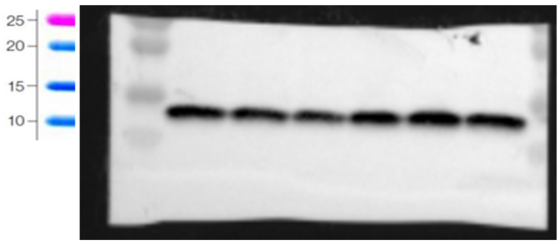

Fig 5C Cyt c

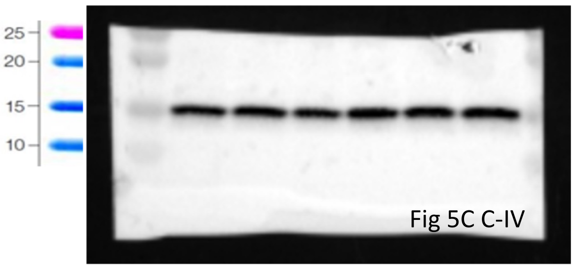

Fig 5C C-IV

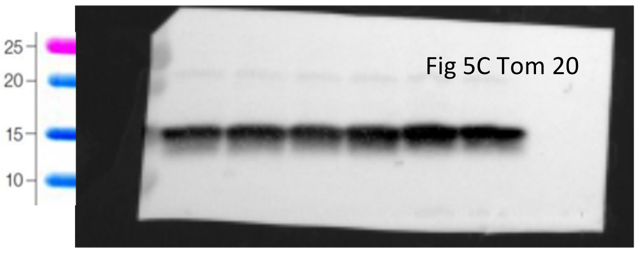

Fig 5C Tom 20

1C
Figure 6B
Original blots

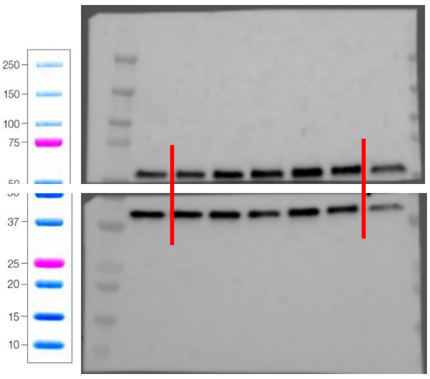

Fig 5A Catalase

Fig 5B β-Actin

Figure S4

Now Figure S5 Original blots

Original WB DATA

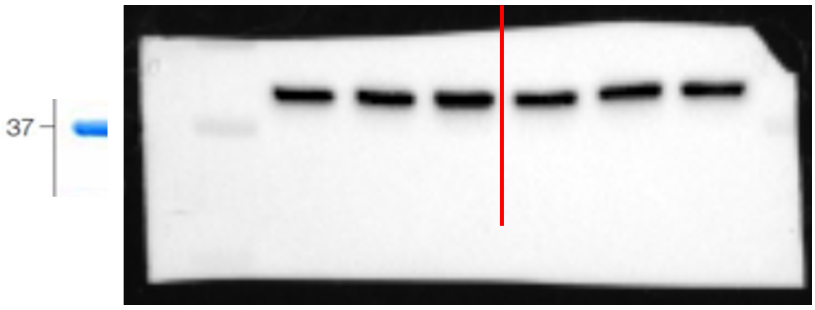

Fig S4 β-Actin

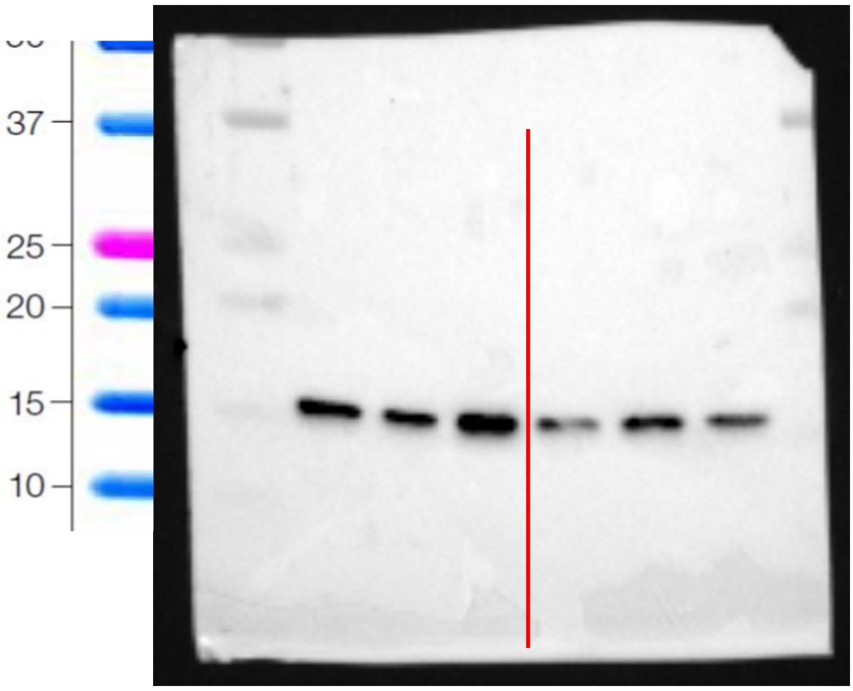

Fig S4 C-IV

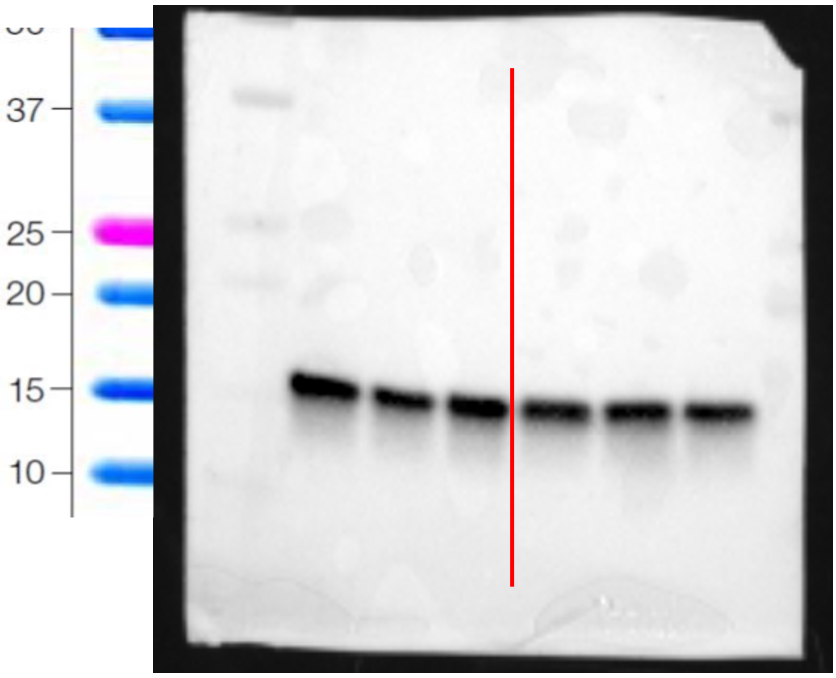

Fig S4 Tom20

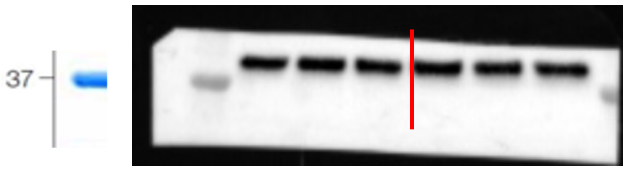

Fig S4 β-Actin

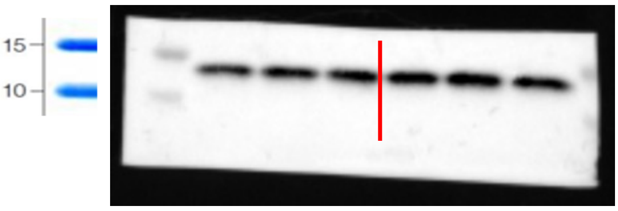

Fig S4 Cyt C

Original WB DATA

Fig. S3b
Original blots

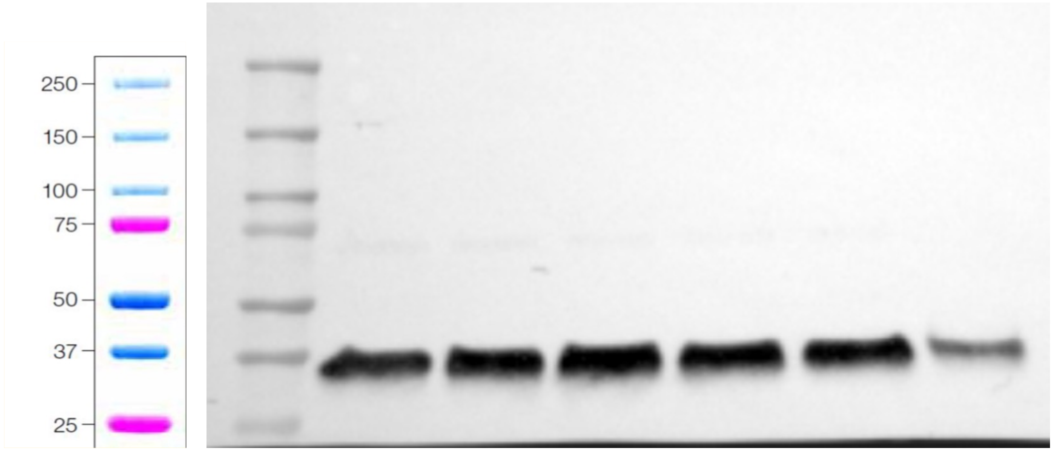

GAPDH
36 KDa

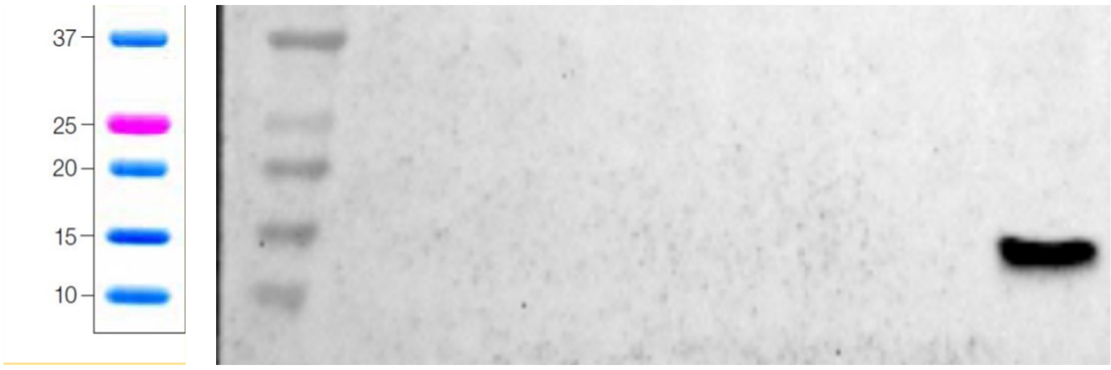

CYT c
14 KDa
